# Mouse monoclonal antibodies against *Clostridioides difficile* toxins TcdA and TcdB target diverse epitopes for neutralization

**DOI:** 10.1128/iai.00139-25

**Published:** 2025-08-22

**Authors:** Heather K. Kroh, Jaime L. Jensen, Sabine Wellnitz, Jeong Jin Park, Alexandre Esadze, Kevin W. Huynh, Mark Ammirati, Seungil Han, Annaliesa S. Anderson, D. Borden Lacy, Alexey Gribenko

**Affiliations:** 1 Department of Pathology, Microbiology, and Immunology, Vanderbilt University Medical Center 12328, Nashville, Tennessee, USA; 2 Vaccine Research and Development, Pfizer Inc 365771 , Pearl River, NewYork, USA; 3 Discovery Sciences, Pfizer Inc 365771 , Groton, Connecticut, USA; 4 Department of Veterans Affairs, Tennessee Valley Healthcare System 20106, Nashville, Tennessee, USA; University of Illinois Chicago, Chicago, Illinois, USA

**Keywords:** monoclonal antibodies, *C. difficile*, epitope mapping, neutralization assays, toxin A, toxin B, functional assays, cryo-EM

## Abstract

*Clostridioides difficile* is a spore-forming, Gram-positive bacterium that can cause infections in subjects with weakened immune system or following antibiotic treatment. These infections may lead to pseudomembranous colitis and antibiotic-associated diarrhea in humans. As such, *C. difficile* is a major cause of nosocomial illness worldwide. Major virulence factors of the bacterium are the large clostridium toxins A (TcdA) and B (TcdB)—high molecular mass proteins with intrinsic glucosyltransferase activity. Toxins bind to the intestinal epithelium and undergo endocytosis by the epithelial cells, followed by a conformational change triggered by the low pH of early endosomes. This conformational change leads to the exposure of hydrophobic segments, followed by membrane insertion, formation of pores, and translocation of the glucosyltransferase domain into the cellular cytoplasm. Once in the cytoplasm, the glucosyltransferase domain inactivates small GTPases of the Rho family of proteins, leading to the disruption of the cytoskeleton. In the current work, we describe the discovery and characterization of a panel of neutralizing mouse monoclonal antibodies capable of interfering with several steps of cellular intoxication by the toxins. The antibodies were produced using hybridoma technology. Neutralizing activity of the antibodies was confirmed using toxin neutralization assays, and functional assays were used to identify specific neutralization mechanisms. Binding epitopes of the antibodies were identified by hydrogen-deuterium exchange mass spectrometry and confirmed through negative-stain and cryo-electron microscopy. Together, our results show that full-length toxins and/or genetically- and chemically-modified toxoids can induce a wide spectrum of antibodies capable of neutralizing the toxins via a variety of mechanisms.

## INTRODUCTION

The growing burden of *Clostridioides difficile* infection (CDI) on healthcare in the United States continues to drive investigation of new strategies to treat or prevent the disease. It is considered an urgent antibiotic-resistant threat by the Centers for Disease Control and Prevention (1), but the main risk for patients stems from prior antibiotic use that disrupts the microbiome and provides a niche for *C. difficile* colonization. The paradox of using antibiotics to treat the CDI, as well as the prevalence of cross-infection in healthcare environments, can set up a cycle of recurrent infection in more susceptible populations. Only one antibody-based therapeutic, the TcdB-specific monoclonal bezlotoxumab (Zinplava), has been clinically approved to prevent recurrence in patients already diagnosed with CDI. No preventative treatments currently exist. Recent Phase III clinical trials have been directed toward improvement of anti-toxin vaccines, with success in reducing disease severity, but not initial infection (2–4).

The symptoms of CDI are instigated primarily through the actions of two large, structurally homologous exotoxins from *C. difficile*, TcdA and TcdB (308 kDa and 270 kDa, respectively). The toxins target the colonic epithelium to produce gastrointestinal symptoms including nausea, diarrhea, and colitis, and the disease can progress to more severe pathologies like toxic megacolon. TcdA and TcdB each contain multiple structural domains that mediate recognition of and uptake by the host cells. At the N-terminus are two domains with enzymatic function: the glucosyltransferase domain (GTD) and the autoprocessing domain (APD). The delivery domain (DD) extends outward from the enzymatic domains and adopts an extended β-sheet structural scaffold that supports a stretch of hydrophobic helices. It is predicted that these helices become restructured with low pH to insert into the membrane to form a pore. For TcdB, the C-terminal portion of the delivery domain also contains the host receptor-binding sites (5–7). It is followed by the C-terminal CROPs (combined repetitive oligopeptide) domain. Upon engagement of cell surface receptors, the toxins enter the cells through endocytosis. The subsequent acidification of the endosome triggers conformational changes in the toxins to form a pore within the endosomal membrane. The pore allows for the delivery of the enzymatic cargos, the GTD and APD, into the host cell cytosol. Activation of the APD by endogenous inositol hexakisphosphate (InsP6) leads to cleavage and release of the GTD, which targets small Rho-family GTPases for glucosylation.

Due to their central role in the symptoms of CDI, TcdA and TcdB are attractive targets for the development of neutralizing antibodies. Examples of human neutralizing monoclonal antibodies (mAb) whose mAb-toxin structures are known include CDA1 (actoxumab), a TcdA-specific mAb that was discovered concurrently with MDX-1388 (bezlotoxumab) (8), and the humanized PA50 (anti-TcdA) and PA41 (anti-TcdB) mAbs (9). Actoxumab and bezlotoxumab were first developed in a human IgG-expressing mouse model, and both recognize the CROPs domains of the toxins (8, 10). PA50 also binds the TcdA CROPs, although it recognizes different repeats than actoxumab (9, 11, 12), with both mAbs neutralizing through aggregation of the toxin. In contrast to these, PA41 binds the TcdB GTD and neutralizes by either preventing pore formation or blocking GTD delivery through the pore (9, 13, 14).

The recent Pfizer clinical trials used a toxoid-based approach, employing genetically modified and chemically inactivated (via 1-ethyl-3-[3-dimethylaminopropyl]carbodiimide hydrochloride/*N*-hydroxysulfosuccinimide (EDC/NHS) treatment) toxins as vaccine antigens (15). A potential caveat in using a toxoid as an antigen is that neutralizing epitopes could be altered during chemical modification, limiting the effective scope of the elicited antibodies. The question of whether a TcdB toxoid can elicit a potently neutralizing antibody response is, in part, the underlying basis for the development of the mAbs within this study.

In this work, we identified and characterized mouse mAbs that were capable of neutralizing wild-type toxins in cell culture from a larger panel of hybridoma-derived antibodies. They were raised against either wild-type TcdA or the toxoid TcdB, with chemical inactivation and genetic point mutations eliminating the glucosyltransferase and proteolytic activities of the toxin. The antibody epitopes were defined through biochemical and structural analyses, including hydrogen-deuterium exchange mass spectrometry, isothermal titration calorimetry, and negative-stain and cryo-electron microscopy. The distribution of neutralizing epitopes across the toxin structural domains suggests that the antibodies neutralize through several different molecular mechanisms. *In vitro* experiments defined the point in the intoxication pathway affected by each mAb, with pore formation emerging as an effective target for inhibition. The patterns of mAb recognition within our panel revealed a diverse repertoire of neutralizing epitopes for both TcdA and TcdB.

## RESULTS AND DISCUSSION

### Binding parameters of the monoclonal antibodies against TcdA and TcdB determined using isothermal titration calorimetry

We employed ITC to determine the binding affinities and enthalpy changes associated with antibody-toxin binding and to compare binding to the wild-type toxin vs vaccine toxoids. The results are shown in Table 1. Most of the mAbs (with the exception of mCDIFA-60-22 and mCDIFB-56-15) bound to their respective toxin/toxoid antigens with low nanomolar affinity and stoichiometries close to 1 Fab fragment bound per antigen, as expected. Binding was driven predominantly by the favorable enthalpy changes. For mCDIFA-60-22, the data suggested a stoichiometry of 6-7 antigen-binding fragments (Fabs) binding to TcdA. The plausible explanation is that the mAb recognizes multiple copies of an epitope in the C-terminal repeat domain. It should be noted that EDC treatment (and/or amino acid substitutions present in the toxoids) had no appreciable effect on antibody binding to either TcdA or TcdB. This observation suggests that chemical and genetic modification of the toxins did not affect the integrity of the epitopes of the mAbs under study.

**TABLE 1:** Monoclonal antibody binding parameters determined by ITC

mAb	Stoichiometry^*a*^^,*b*^		*K*_d_ (nM)^*a*^^,*b*^		Δ*H*, kcal/^mol*a**,^b^*^	
	Toxin	Toxoid	Toxin	Toxoid	Toxin	Toxoid
mCDIFA-248-25	1.07 ± 0.01	0.96 ± 0.01	1 ± 1	2 ± 1	−15.5 ± 0.3	−20.3 ± 0.2
mCDIFA-184-9	1.15 ± 0.01	1.06 ± 0.01	4 ± 1	6 ± 3	−19.4 ± 0.3	−19.7 ± 0.3
mCDIFA-205-7	1.31 ± 0.02	1.18 ± 0.01	38 ± 25	2 ± 1	−1.4 ± 0.1	−6.3 ± 0.3
mCDIFA-230-2	1.28 ± 0.01	1.47 ± 0.03	90 ± 22	9 ± 2	−4.1 ± 0.1	−11.0 ± 0.3
mCDIFA-60-22	6.16 ± 0.06	6.18 ± 0.11	32 ± 4	7 ± 5	−13.2 ± 0.3	−16.1 ± 0.6
mCDIFB-6-30	1.05 ± 0.01	0.83 ± 0.01	<1	2 ± 1	−24.0 ± 0.2	−26.0 ± 0.2
mCDIFB-8-26	1.25 ± 0.01	0.91 ± 0.01	2 ± 1	9 ± 2	−13.7 ± 0.2	−20.3 ± 0.4
mCDIFB-56-15	1.11 ± 0.01	1.06 ± 0.01	38 ± 14	69 ± 13	−4.4 ± 0.2	−4.9 ± 0.1

^*a*^ Both toxins and corresponding toxoids were titrated with the antibodies to check if chemical and genetic modifications may have affected antibody binding epitopes.

^*b*^ Reported errors (“±”) are errors of the fit to the “single class of binding sites” model. *K*_d_, equilibrium dissociation constant; Δ*H,* enthalpy change upon binding.

### Measurement of the functional toxin neutralizing activity of the different *C. difficile* anti-toxin mAbs

**Fig 1:**
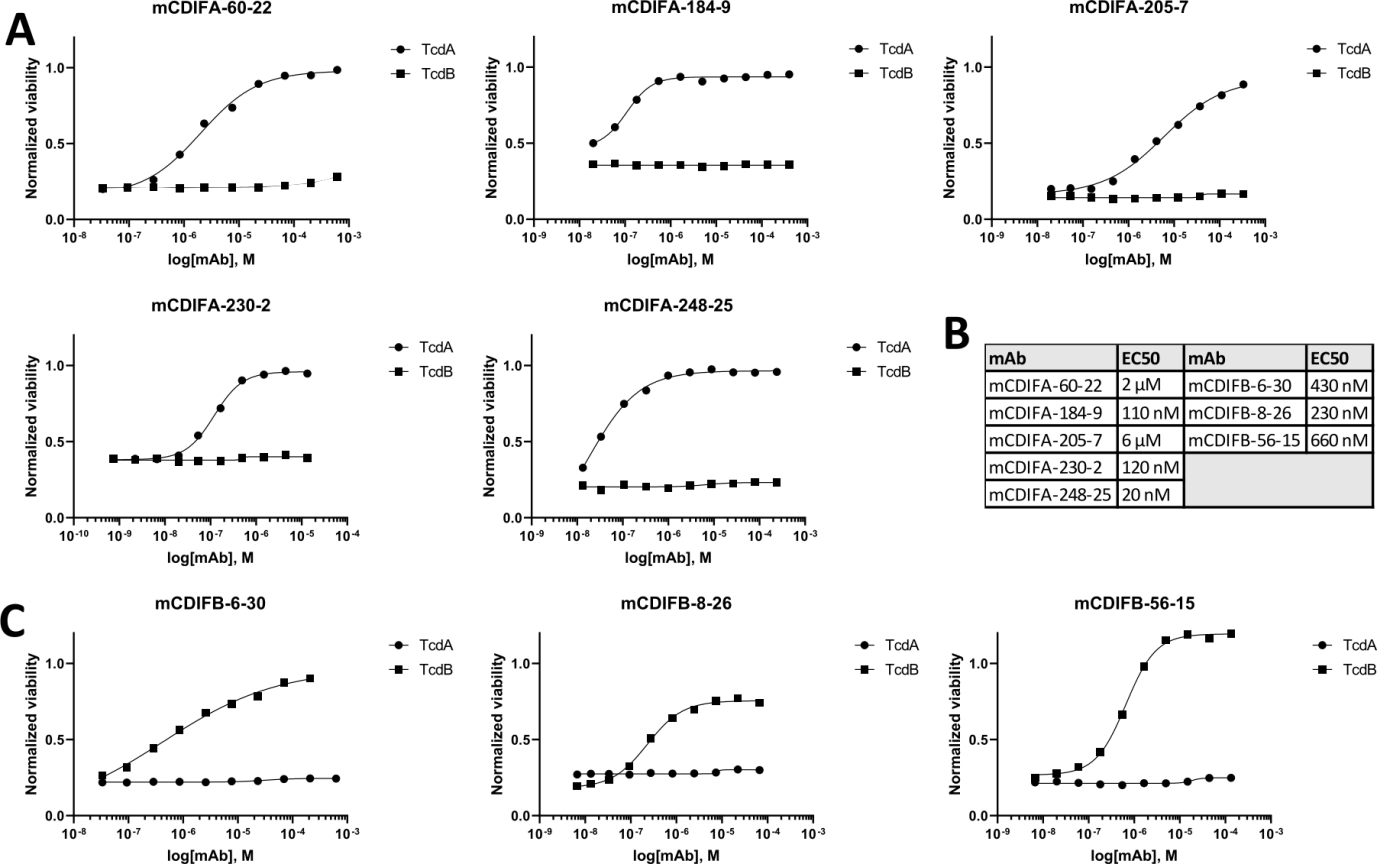
Neutralization of TcdA or TcdB by antibodies on Vero cells. (**A**) Neutralization results for TcdA with mAbs. (**B**) EC50 results from analysis of neutralization data. (**C**) Neutralization results for TcdB with mAbs. Technical replicates were averaged for analysis in GraphPad Prism with least squares fit of the model.

A toxin neutralization assay (TNA) was optimized with Vero cells to assess the neutralization capacity of each antibody. TcdA or TcdB was applied to the cells in the presence of antibodies at multiple concentrations, and the viability of the cells was quantified 72 h after addition of toxin-antibody solution to cells using ATP luminescence. The luminescence values were normalized to a standard curve obtained with a defined pool of human sera with low, medium, and high anti-*C. difficile* toxin antibody titers, to compare the values to the toxin-only and no toxin controls. This analysis allowed for the estimation of each antibody’s EC_50_ value. Representative neutralization curves are shown and indicate that TcdA- and TcdB-specific mAbs are capable of neutralizing, albeit at different potencies (Fig. 1A through C).

Comparing our toxin neutralization data with bezlotoxumab, it appears that our TcdB-specific antibodies have slightly lower neutralization potential (EC_50_ of 230–660 nM [Fig. 1] vs 10–100 nM for bezlotoxumab [10]). It should be noted, however, that none of the TcdB-specific antibodies reported in this manuscript recognize the CROPs domain of the toxin. Three of the five TcdA-specific antibodies described in the current work have neutralization potential comparable to that of bezlotoxumab (Fig. 1).

### Impact of monoclonal antibodies on the ability of toxins to bind to the cell surface

The first step in host cell intoxication by TcdA or TcdB requires binding of the toxin to the cell surface through toxin-specific receptors. Neutralizing antibodies can obstruct this process either through direct blockade of a receptor binding site on the toxin or, less specifically, through aggregation of the toxin-antibody complexes. Both mechanisms would reduce functional binding and uptake into the cell. Cell-binding experiments were used to assess whether the neutralizing mAbs or their corresponding Fabs could significantly reduce toxin binding. Since the toxin receptor profiles differ between cell types, we performed the studies in three epithelial cell lines: Caco-2 (human colon), Vero (simian kidney), and A549 (human lung; TcdA was not tested on this cell line due to technical difficulties).

**Fig 2:**
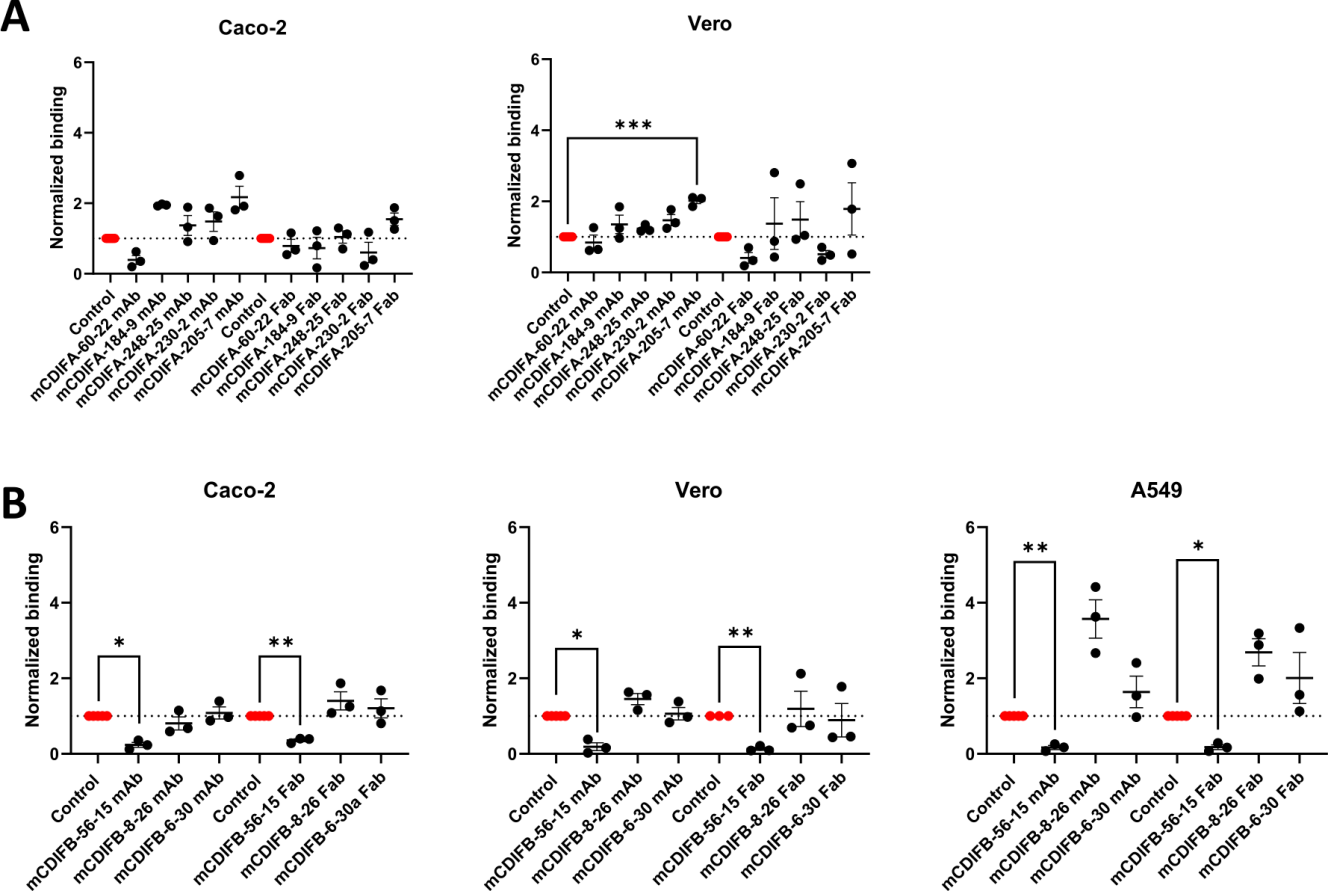
Effect of mouse neutralizing antibodies on binding of TcdA and TcdB. Panels show binding of TcdA to Caco-2 and Vero cells (A), or TcdB to Caco-2, Vero, and A549 cells (B) after treatment with individual TcdA- and TcdB-specific mAbs and Fabs. Toxin-only controls are shown in red, and all antibody-containing reactions are normalized to their corresponding controls. Assays were performed in triplicate and analyzed in GraphPad Prism by one-way ANOVA using Dunnett’s multiple comparisons test. (*P* * <0.05, ** <0.01, *** 0.0001)

For TcdA, experiments on Caco-2 and Vero cells showed no significant reduction in toxin binding with either the mAbs or Fabs, with the exception of mCDIFA-205-7 which did significantly reduce binding in Vero cells compared to the control (Fig. 2A and B). In contrast, a striking reduction in bound TcdB was observed with both the mAb and Fab of mCDIFB-56-15 on Caco-2, Vero, and A549 cells (Fig. 2C through E). The other two TcdB-specific antibodies showed either no change in, or modestly increased, binding to cells. This result supports a neutralization mechanism for mCDIFB-56-15 of direct blockade of toxin binding to the cell surface by the antibody. Notably, results of the HDX and negative stain EM experiments with this mAb (described below) place the toxin-antibody interface near the proposed CSPG4-binding site (6).

### Reduction in toxin-mediated cellular depolarization by monoclonal antibodies

The molecular events governing the insertion of TcdA or TcdB into cellular membranes to form functional pores have not been defined. Previous studies have used the release of radioactive rubidium from CHO cells as a reporter of membrane permeabilization (16, 17). This technique relies on a low pH environment to induce conformational changes within the toxins and subsequent pore formation at the cell surface, but it reports ion movement in one direction only: out of the cells. Membrane permeabilization in response to the toxins could trigger movement of ions in either direction, which would lead to either hypo- or hyper-polarization of the intoxicated cell. The associated changes in cell polarity can be monitored with fluorescent, membrane-specific dyes that report fluctuations in membrane potential. The DiBAC (Bis-(1,3-Dibutylbarbituric Acid)Trimethine Oxonol) dyes are classified as slow-response reporters and are appropriate for changes on the seconds to minutes time scale (18, 19). They bind to lipid membranes, then migrate and redistribute within the cell during hypopolarization, where the quantum yield of the dye increases in the lower-potential environment. We have found that the resulting change in fluorescence of DiBAC_4_(3) can be measured kinetically to monitor the toxins’ effects on cells, under both neutral and acidic conditions.

**Fig 3:**
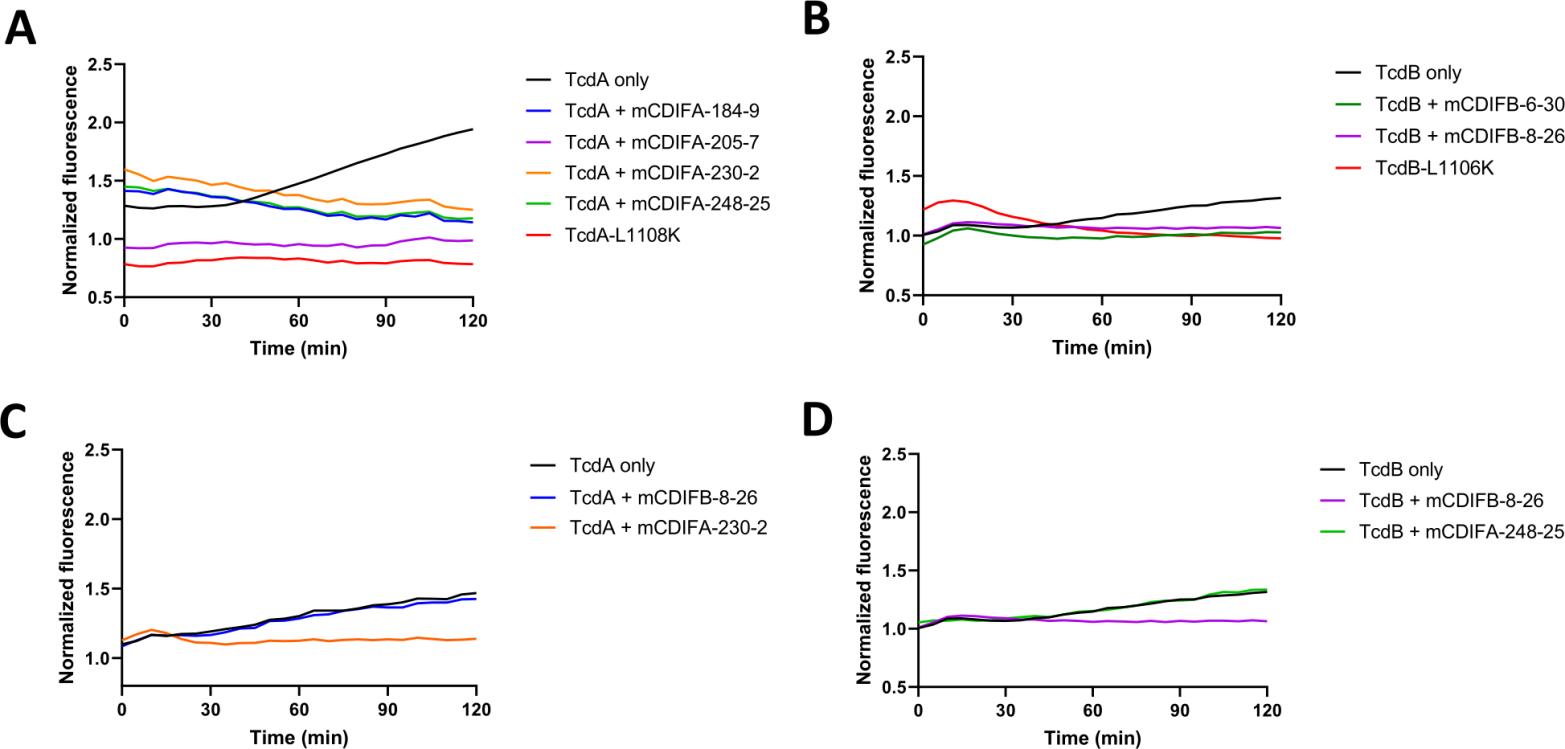
Antibodies protect human epithelial cell lines from depolarization by TcdA (HT29 cells, pH 5.0) or TcdB (A549 cells, pH 7.2), measured by changes in DiBAC4(3) fluorescence. (**A**) TcdA (10 nM, black) with 10 nM of mCDIFA-184-9 (blue), or mCDIFA-205-7 (purple), mCDIFA-230-2 (orange), or mCDIFA-248-25 (green). TcdA-L1108K (pore-formation mutant) is shown in red. (**B**) TcdB (1 nM, black) with 10 nM of mCDIFB-6-30 (green) or mCDIFB-8-26 (purple). TcdB-L1106K is in red. mCDIFA-60-22 and mCDIFB-56-15 were not included, since they block binding of the cell surface. Unrelated antibodies do not protect from depolarization, as shown in controls comparing (**C**) TcdA (10 nM, black) with 10 nM of mCDIFB-8-26 (blue) or mCDIFA-230-2 (orange), and (**D**) TcdB (1 nM, black) with 10 nM of mCDIFA-248-25 (green) or mCDIFB-8-26 (purple). Fluorescence is normalized to a no-toxin control, and all traces represent the average of three independent replicates.

Previous studies have demonstrated pore formation by TcdA at acidic pH on HT29 cells (17, 20). Increased DiBAC_4_(3) fluorescence was observed in these cells in the presence of TcdA at pH 5.0 (Fig. 3A), but not at neutral pH (pH 7.2) and not in the presence of TcdA_L1108K,_ a defined pore-forming mutant (21). All four TcdA-specific antibodies that recognize the delivery domain (mCDIFA-248-25, mCDIFA-230-2, mCDIFA-184-9, and mCDIFA-205-7) blocked the increase in fluorescence seen with 10 nM TcdA alone at acidic pH (Fig. 3A), but the TcdB-specific mCDIFB 8-26 did not (Fig. 3C).

In contrast to the low pH TcdA experiments, we saw depolarization of A549 cells in response to TcdB at both acidic and neutral pH. The depolarization was not observed with the TcdB_L1106K_ pore-forming mutant. Increased DiBAC_4_(3) fluorescence can be detected at 1 nM TcdB at neutral pH in A549 cells (Fig. 3B). Both mCDIFB-8-26 or mCDIFB-6-30 mAb prevented the increase in fluorescence (Fig. 3B), with no reduction seen with mCDIFA-248-25 (Fig. 3D). The mAbs mCDIFB-8-26 and mCDIFB-6-30 target different epitopes on TcdB GTD. Their ability to block depolarization may indicate a neutralization mechanism akin to PA41, preventing GTD cargo delivery into cells.

### Protection from toxin-mediated glucosylation of the GTPase Rac1 by monoclonal antibodies

**Fig 4:**
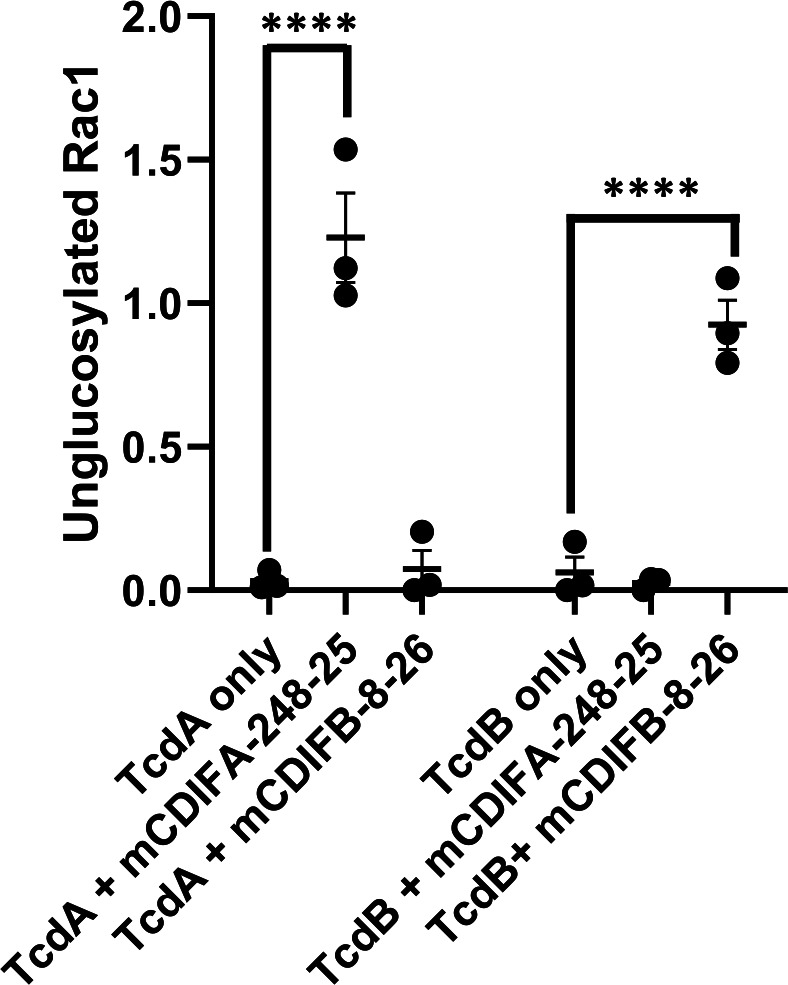
Protection of Rac1 from toxin-mediated glucosylation in Caco-2 cells. TcdA (10 nM) or TcdB (10 nM) were pre-incubated with 10-fold molar excess of mAb before cell intoxication. Results are normalized to total Rac1 and represent three independent replicates (*P* **** <0.0001).

The intoxication pathway culminates in the release of the GTD from the toxins and modification of the host cell Rho-family GTPases through glucosylation. The most commonly tested target for both TcdA and TcdB is Rac1, which shows significant modification post-intoxication (22). Any inhibition of upstream events during intoxication would result in protection of the GTPase. Western blotting of cell lysates was used to measure GTPase glucosylation and determine if TcdA- and TcdB-specific mAbs that prevent depolarization also protect Rac1. Caco-2 cells were intoxicated with TcdA or TcdB in the presence of either mCDIFA-248-25 or mCDIFB-8-26, and levels of unglucosylated and total Rac1 were compared to assess protection afforded by the antibodies. Expectedly, TcdA-specific mCDIFA-248-25 protects Rac1 from modification by TcdA only, with mCDIFB-8-26 only exhibiting protection during the exposure of Caco-2 to TcdB (Fig. 4).

### Mapping the antibody-binding epitopes using hydrogen-deuterium exchange-mass spectrometry

To gain further understanding of neutralization mechanisms, we have mapped antibody-binding epitopes using HDX-MS. Levels of deuterium uptake in each proteolytic peptide derived from the antigen alone vs corresponding peptide derived from the protein-antibody complex were compared at several exchange time points. A decrease in deuterium incorporation indicates protection of those specific toxin residues in the presence of the antibody (Fig. S1 through S8). Peptide pairs with identical levels of deuteration in the presence and absence of antibody were presumed to fall outside of the antibody-binding epitope. Epitope segments (amino acids) were assigned for each toxin-antibody interaction and are listed in Table 2. It should be noted that the reported results do not suggest that each amino acid within the segment interacts with the antibody, only that the interacting residues are located within these segments.

**TABLE 2:** Epitope segment assignment for toxoid-antibody complexes determined by hydrogen-deuterium exchange mass spectrometry

mAb	Segment 1	Segment 2	Segment 3
mCDIFA-60-22	2,309–2,386	2,479–2,499	2,660–2,673
mCDIFA-184-9	1,069–1,093	1,462–1,475	–
mCDIFA-205-7	1,109–1,114	1,329–1,337	1,355–1,359
mCDIFA-248-25	1,126–1,144	1,206–1,212	1,245–1,254
mCDIFA-230-2	1,242–1,254	–^*a*^	–
mCDIFB-6-30	297–307	346–360	–
mCDIFB-8-26	18–30	–	–
mCDIFB-56-15	1,704–1,708	1,730–1,740	1,783–1,796

^*a*^ –, no changes in deuteirum uptake.

### TcdA-specific antibodies

#### mCDIFA-60-22

Multiple peptides distributed through the TcdA CROPs were found to have consistent decreases in deuterium uptake (Fig. S1). Correlation of these HDX results with the structural organization of the CROPs domain (as described by Ho et al. [23]) is shown in Table 3. The CROPs domain is arranged in what is defined here as “modules,” consisting of 3–5 short repeats, followed by a long repeat. A total of 8 modules are found within the CROPs domain of TcdA (with the last module formed by amino acid residues 2,645–2,710 lacking a long repeat). HDX data indicate that residues comprising short repeats 1–4 from the repeat module V, parts of repeats 2–3 from the repeat module VI, and parts of repeats 1–2 from the repeat module VIII are involved in mCDIFA-60-22 binding. Close examination of the sequences points to a conserved motif (A(A/V)TGWQTI(D/N)GK(K/V)YYF) found in all of these amino acid segments. There are three copies of this motif in the repeat module V and one copy in each of the repeat modules VI and VIII. Furthermore, two more copies of this motif are found in the repeat module III (Table 3) although no decrease in deuterium uptake within repeat module III was observed in the HDX experiments. This could be due to undersaturation of the antigen (a 4:1 ratio of Fabs to the antigen was used in the HDX-MS runs). Taken together, the number of mCDIFA-60-22 epitopes found in the CROP domain could be as high as seven, in good agreement with the ITC results, which revealed a stoichiometry of between 6 and 7 mCDIFA-60-22 Fab fragments bound per each toxin molecule (epitope localization and binding stoichiometry suggest a potential mechanism of toxin neutralization by the antibody). The presence of multiple copies of the epitope is expected to lead to the formation of large immunocomplexes due to the crosslinking of multiple epitopes on different toxin molecules by the antibodies, resulting in toxin precipitation. This was, in fact, observed *in vitro,* which is why Fab fragments had to be used in the HDX-MS experiments.

**TABLE 3:** mCDIFA-60-22 epitope mapping results correlate to the modular organization of the TcdA CROPs domain (23)

Repeat module	Short repeat number	Amino acid Sequence^*a*^
**III**	2 3 4	2079–2099 GLQTIDSKKYYFNTNTAE*AAT* 2100–2120 *GWQTIDGKKYYF*NTNTAE*AAT* 2121–2141 *GWQTIDGKKYYF*NTNTAIAST
**V**	1 2 3 4	2307–2326 KFLTLNGKKYYFDNDSK*AVT* 2327–2347 *GWQTIDGKKYYF*NLNTAE*AAT* 2348–2368 *GWQTIDGKKYYF*NLNTAE*AAT* 2369–2389 *GWQTIDGKKYYF*NTNTFIAST
**VI**	2 3	2460–2481 GLRTIDGKKYYFNTNTAV*AVT* 2482–2502 *GWQTINGKKYYF*NTNTSIAST
**VIII**	1 2	2645–2664 RFLHLLGKIYYFGNNSK*AVT* 2665–2686 *GWQTINGKVYYF*MPDTAMAAAG

^*a*^ Conserved sequence motifs showing decreased deuterium uptake in the presence of mCDIFA-60-22 Fabs is italicized and underlined in each repeat sequence.

#### mCDIFA-184-9

**Fig 5:**
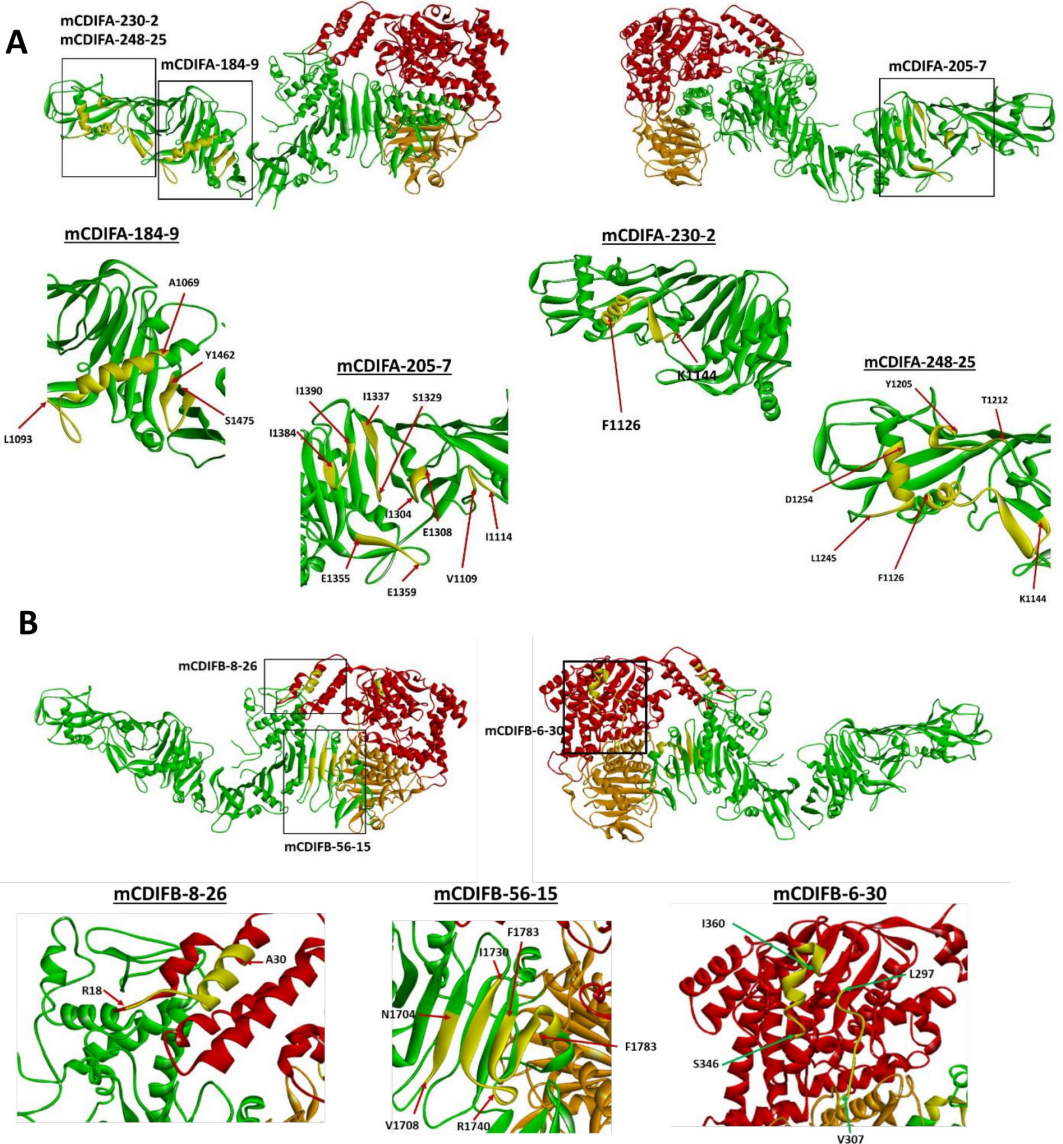
Binding epitopes of TxdA- and TcdB-specific monoclonal antibodies mapped onto the three-dimensional structures of the toxins. (**A**) Three-dimensional structure of TcdA (4R04.pdb [5]) with identified monoclonal antibody bound epitopes illustrated. Red—N-terminal (glucosyltransferase) domain, orange—proteolytic domain, green—delivery domain. (**B**) Three-dimensional structure of TcdB (6OQ5.pdb [17]) with identified monoclonal antibody bound epitopes illustrated. Red—N-terminal (glucosyltransferase) domain, orange—proteolytic domain, green—delivery domain. Sequence segments showing decreased deuterium uptake in the presence of the antibodies are shown in yellow. Starting and ending residues of each segment are indicated in the expanded views. Structural illustrations were rendered using Discovery Studio Visualizer 4.5.

The HDX data (Fig. S2) indicate that the mCDIFA-184-9 epitope is discontinuous, with epitope segments located between residues 1069–1093 and 1462–1475. Mapping these residues onto the three-dimensional structure of TcdA (4R04.pdb [21]) shows that they are, in fact, adjacent in space (Fig. 5A). Interestingly, the first segment includes residues 1,069–1,083, which form a significant part of the hydrophobic helix involved in translocation pore formation (21), facilitating GTD translocation into the cytosol (24). Residues 1,462–1,475 form a β-hairpin within the underlying “scaffold” structure, which prevents premature aggregation of the hydrophobic helices (21). It is tempting to speculate that mCDIFA-184-9 neutralizes the toxin by tethering the hydrophobic helical segment to the scaffold, preventing release of the helices and, therefore, interfering with toxin pore formation. This proposed structural mechanism explains how antibody mCDIFA-184-9 prevents membrane depolarization and the corresponding increase in DiBAC_4_(3) fluorescence (Fig. 3A).

#### mCDIFA-205-7

Deuterium uptake plots used to identify sequence segments protected by the mAb are shown in Fig. S3. Mapping the assigned segments onto the three-dimensional structure of the toxin shows that these residues form several loops and a β-sheet that are adjacent in space (Fig. 5A). Furthermore, residues 1,109–1,114 are within the hydrophobic segment formed by residues 958–1,130, while residues between 1,304 and 1,390 are within the β-sheet scaffold. Similar to mCDIFA-184-9, we propose that mCDIFA-205-7 neutralizes the toxin by preventing release of the hydrophobic helices and, correspondingly, preventing TcdA pore formation, in agreement with experimental results shown in Fig. 3A.

#### mCDIFA-248-25

Deuterium uptake plots used to identify sequence segments protected by the mAb are shown in Fig. S5. Mapping the assigned residues on the structure (1126-FNHLSESKKYGPLKTEDDK-1144, 1206-SAIGIET-1212, 1245-LENDGTRLLD-1254, Fig. 5A) suggests that the first segment is not likely to extend all the way to residue 1144 and probably ends at or near residues G1136-P1137;, however, resolution of the current proteolytic digest map is not sufficiently high to confirm this observation. The data indicate that mCDIFA-248-25 recognizes a conformational epitope consisting of three structural segments at the distal end of the delivery domain of the toxin. Similar to mCDIFA-184-9, the first segment of the identified epitope includes the very end of the hydrophobic helical stretch (21) thought to be involved in pore formation (24), while the other two segments are parts of the underlying scaffold structure (21). Localization of the structural elements suggests that mCDIFA-248-25 also blocks helix release from the scaffold and pore formation, in line with data shown in Fig. 3A. To note, the analysis for another antibody, mCDIFA-230-2, showed possible protection in the same peptide regions as mCDIFA-248-25 (Fig. 5A; Fig. S4), but it also displayed poor overall data quality. The two antibodies are expected to have similar epitopes.

### TcdB-specific antibodies

#### mCDIFB-6-30

HDX-MS data (Fig. S6) suggested that deuterium uptake in the presence of the antibody is decreased between residues 297–307 and 346–360. Mapping the peptides covering these residues on the three-dimensional structure of TcdB shows that a continuous surface could be formed by residues 297–301 and 346–360 (Fig. 5B), potentially further narrowing down the epitope. We do not, however, have experimental HDX data that would allow us to say with confidence that residues 302–307 are not involved in the antibody binding. A neutralizing antibody PA41 reported by Kroh et al. (13) recognizes an epitope that overlaps with that of mCDIFB-6-30; both mCDIFB-6-30 and PA41 bind to the helix formed by residues 340–351 (although the extent of binding may be somewhat different). Unlike mCDIFB-6-30, PA41 also binds to residues 322–325, that are on the opposite side of that helix from residues 297–301 recognized by mCDIFB-6-30. PA41 blocks delivery of the glycosyltransferase domain into the cells. It is reasonable to suggest that the neutralization mechanism of mCDIFB-6-30 is like that of PA41 since their epitopes overlap.

#### mCDIFB-8-26

The HDX data (Fig. S7) suggested that the mCDIFB-8-26 binding epitope is within the segment formed by residues 18–30 (residues 29–30 are included in the epitope assignment, since peptides starting at residue 29 “report” on residues 31 and beyond (25). Mapping these residues to the 3D structure of the antigen shows that they form a significant part of the second N-terminal helix (Fig. 5B), which is thought to be involved in cellular membrane targeting of other large clostridial toxins (26–28). Binding of an antibody to this epitope could, therefore, prevent membrane targeting of the glucosyltransferase domain, providing a potential explanation for the mechanism of neutralization. Indeed, we showed that mCDIFB-8-26 is capable of protecting Rac1 from glucosylation in Caco-2 cells (Fig. 4).

#### mCDIFB-56-15

The HDX data (Fig. S8) suggested that the epitope includes residues 1704–1708, 1730–1740, and 1783–1796. Mapping these residues onto the 3D structure of the toxin shows that the residues form a continuous surface across three β-strands of the C-terminal beta-sheet (Fig. 5B). Several reports suggest that this region is critical for TcdB activity, presumably through controlling interaction with the cell targets (29–32). In fact, the deletion of residues 1756–1852, which contain part of the epitope, eliminates toxicity altogether (32). Furthermore, the binding site for one of the known TcdB receptors, CSPG4 (chondroitin sulfate proteoglycan 4), is in close proximity to these residues (6). We observed a significant reduction in toxin B binding to all tested cell lines in the presence of this mAb (Fig. 2B), indicating that interference with the receptor binding is likely the dominant toxin neutralization mechanism afforded by mCDIFB-56-15.

### Epitope visualization by negative-stain electron microscopy of toxin-Fab complexes

**Fig 6:**
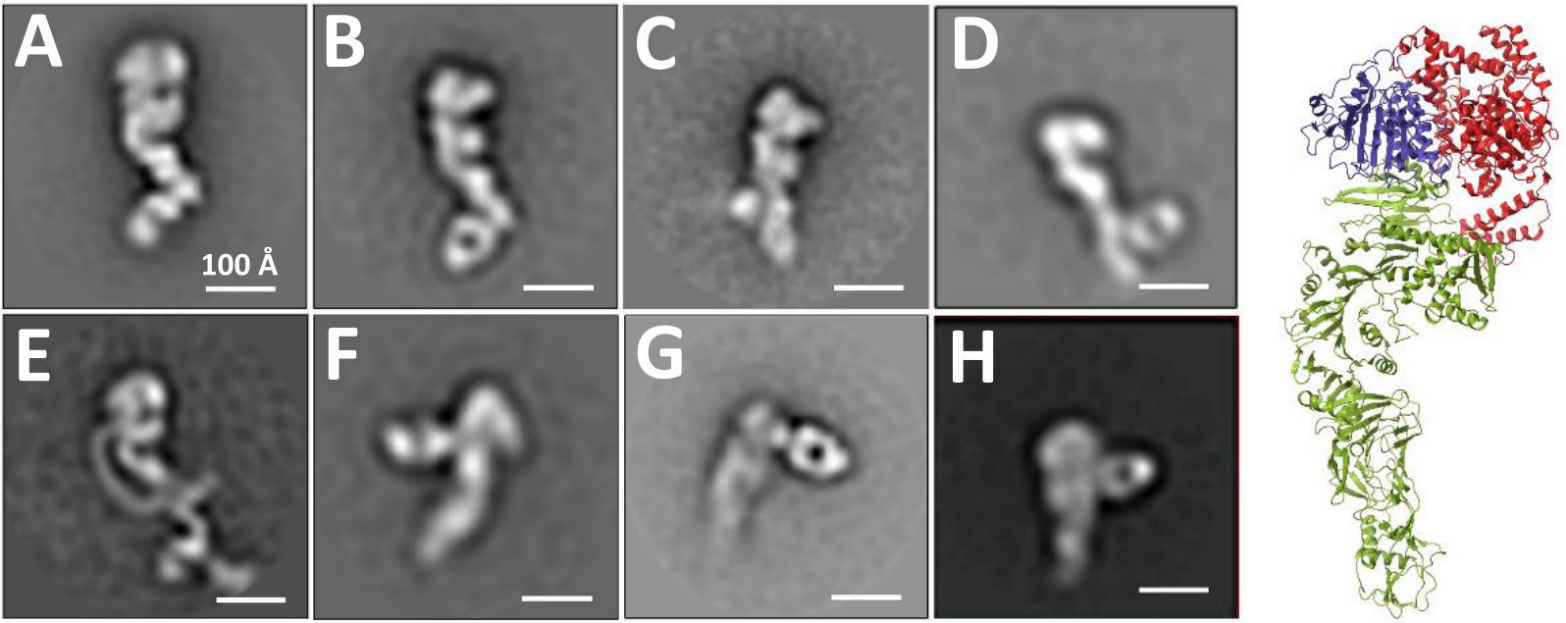
Toxin-antibody Fab complexes visualized by negative-stain electron microscopy. TcdA1-1832 with (**A**) mCDIFA-248-25, (**B**) mCDIFA-230-2, (**C**) mCDIFA-184-9, or (**D**) mCDIFA-205-7. Full length TcdA with (**E**) mCDIFA-60-22. TcdB1-1810 with (**F**) mCDIFB-8-26, (**G**) mCDIFB-6-30, or (**H**) mCDIFB-56-15. Each image is a single class average with a representative view of the complex (scale bars 100 Å). (Right) Crystal structure of TcdA1-1832 (4R04.pdb) with the GTD in red, the auto-processing domain in blue, and the delivery domain in green.

To confirm the HDX results and directly visualize the antibodies bound to the toxins, antibody Fab fragments were generated by proteolytic digestion and incubated with TcdA and TcdB for negative-stain electron microscopy. Toxin truncations (TcdA_1–1832_ or TcdB_1–1810_) were used to eliminate the conformational heterogeneity associated with the CROPs domain, but the mCDIFA-60-22 Fab was visualized in complex with full-length TcdA due to its binding to multiple repeats within the CROPs domain. Particle data sets were collected for each sample and aligned to generate two-dimensional class averages at low resolution (15–20 Å) (Fig. 6). In each case, the Fab was observed clearly bound in the approximate location predicted by the HDX-MS data.

mCDIFA-248-25 and mCDIFA-230-2 recognize similar epitopes on the tip of the TcdA delivery domain, but the Fabs appear bound at slightly different angles relative to the delivery domain (Fig. 6A and B). The mCDIFA-184-9 Fab is found near the center of the TcdA delivery domain (Fig. 6C), and mCDIF-205-7 is located on the opposite side of this domain compared to the position of the other Fabs (Fig. 6D).

mCDIFA-60-22 occupies multiple sites along the TcdA-CROPs, most likely at repeats 5/6/7 (Fig. 6E), consistent with the pattern of sequence conservation between the repeats. Recognition of multiple CROPs repeats has been observed with the other neutralizing mAbs against TcdA; PA50 has a similar pattern of recognition for the CROPs as mCDIFA-60-22, and actoxumab binds at repeats 3 and 5 (9, 11, 12). Earlier studies characterizing TcdA CROPs-specific nanobodies identified modest neutralizers against repeats 5/6/7 (16).

Complexes with the two TcdB GTD-specific Fabs, mCDIFB-8-26 and mCDIFB-6-30a, also produced well-aligned classes. mCDIFB-8-26 is near the GTD of TcdB, extending at an approximately 90 degree angle from the main body of the toxin, consistent with the location of the membrane localization domain (MLD) (Fig. 6F). In contrast, mCDIFB-6-30, which has an epitope similar to that of PA41 (13), shows the Fab angled out from the end of TcdB (Fig. 6G), corresponding to the opposite face of the GTD from mCDIFB-8-26. Based on the HDX data, mCDIFB-56-15 targets a unique epitope near the “hinge” between the delivery and CROPs domains, and the Fab is observed bound to this region (Fig. 6H). The potential steric hindrance imparted by a bound antibody near the CSPG4-binding site in this region of TcdB could be central to its ability to neutralize the toxin.

### Cryo-electron structure of TcdA in complex with mCDIFA-248-25 Fab

**Fig 7:**
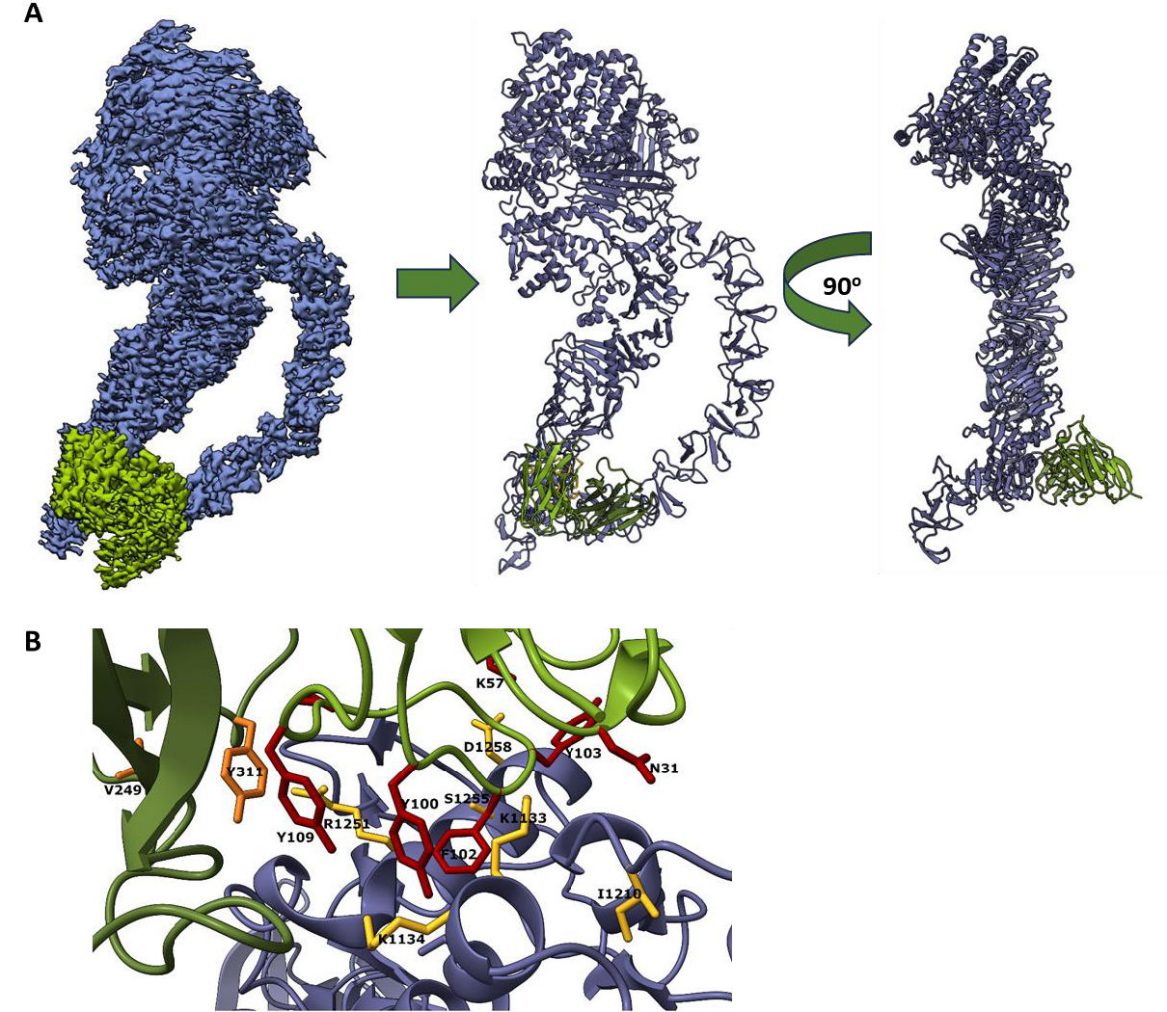
Cryo-electron microscopy of TcdA in complex with mCDIF248-25 Fab. (**A**) Model of complex (3.23 Å resolution), rotated 90 degrees to visualize Fab orientation at the end of the TcdA delivery domain (TcdA blue, mCDIF248-25 Fab green). (**B**) Residues involved in hydrogen bonding in the toxin-antibody epitope are highlighted in yellow (TcdA), red (Fab heavy chain), and orange (Fab light chain).

To achieve a higher-resolution view of a representative mAb against the TcdA delivery domain, the Fab fragment of mCDIFA-248-25 was complexed with full-length TcdA for cryo-electron microscopy (Fig. 7). The resulting 3.23 Å structure contains nearly all of TcdA, with only the most C-terminal repeats of the CROPs (repeats 6–7) unresolved (Fig. 7A). The mCDIFA-248-25 Fab interacts directly with the cluster of helices near the tip of the delivery domain, predominantly with the helix containing residues 1246–1260 and portions of the helices 1118–1135 and 1204–1208 (Fig. 7B). The residues contained within the epitope are consistent with the pattern of protection shown by HDX-MS (Fig. 5A; Fig. S4); several other protected TcdA residues identified by HDX-MS are located adjacent to those involved in hydrogen bonds in the structure. The Fab heavy chain contributes several hydrophobic residues to the paratope (Tyr103, Phe102, Tyr100, Tyr109), with the Fab light chain having a minor role that is mostly mediated by Tyr311 (Fig. 7B). In addition, one salt bridge is found between Fab heavy chain Lys57 and Asp1258 from TcdA (Fig. 7B). The antibody paratope residues are oriented linearly between the TcdA helices in the epitope; mAb binding and stabilizing this location could prevent conformational changes necessary for unfolding of the helical cluster during formation of a toxin pore.

In summary, we have isolated and characterized mouse mAbs that can neutralize TcdA and TcdB function by binding diverse epitopes. Table 4 summarizes the proposed mechanisms of neutralization for the antibodies within this study. Only two appear to affect initial host cell recognition and binding by the toxins *in vitro*, either through the observed aggregation of the toxin antigen (mCDIFA-60-22) or by potentially blocking one of the identified receptor-binding sites on TcdB (mCDIFB-56-15). Remarkably, the predominant mechanism of neutralization within this panel is consistent with reduction or obstruction of membrane permeabilization by the toxins. For TcdA, the ability of the remaining mAbs to prevent depolarization of intoxicated cells under low pH conditions suggests that they constrain parts of the toxin structure essential to the formation of a conductive pore within the endosomal compartment. The HDX data and the cryo-EM structure highlight several hydrophobic helices within the epitopes that have been associated with pore formation (17).

**TABLE 4:** Summary of proposed neutralization mechanisms for the mouse monoclonal antibodies

Target	Antibody	Toxin epitope	Supporting data	Mechanistic step
TcdA	mCDIFA-248-25	Translocation/delivery	Prevents depolarization at low pH	Pore formation
			Protects Rac1 from modification	
	mCDIFA-230-2	Translocation/delivery	Prevents depolarization at low pH	Pore formation
	mCDIFA-184-9	Translocation/delivery	Prevents depolarization at low pH	Pore formation
	mCDIFA-205-7	Translocation/delivery	Prevents depolarization at low pH	Pore formation
	mCDIFA-60-22	CROPs	Aggregates toxin *in vitro* Multiple epitope recognition	Cell surface binding
TcdB	mCDIFB-8-26	GTD (MLD)	Prevents depolarization at neutral pH	Pore formation or membrane recognition
			Protects Rac1 from modification	
	mCDIFB-6-30	GTD	Prevents depolarization at neutral pH	Pore formation or membrane recognition
	mCDIFB-56-51	Translocation/delivery	Blocks binding of toxin to cells	Cell surface binding

Interpretation of the depolarization data for the TcdB-specific mAbs is less direct, as these two mAbs recognize epitopes on the GTD, not the delivery domain. TcdB with the GTD deleted has been shown to still form pores in the low pH rubidium release assay (17). The events preceding the cellular depolarization observed here under neutral pH have not yet been characterized, as the formation of conductive pores by the toxins has been thought to only occur in the endosome. These events could result from disruption or permeabilization of the phospholipid membrane through direct receptor or membrane engagement mediated by the GTD. The relatively high concentrations of TcdB (>1 nM) needed to induce measurable depolarization under neutral conditions may reflect toxin self-association or ligation of low- abundance receptors. The binding site for mCDIFB-6-30 is similar to that of PA41, which has been proposed to block the delivery of the GTD into the host cell, by preventing either pore formation or delivery of the cargo itself through the pore (13). The mCDIFB-8-26 epitope is located within the TcdB membrane localization domain (MLD), a four-helix bundle at the N-terminus of the toxin; when specific residues in the MLD are mutated, cellular recognition by the toxin is impacted (26, 33). If a bound antibody were to block exposure of these residues, the membrane-specific interactions mediated by this domain could be affected.

Several previous studies have aimed to develop neutralizing mAbs with focused epitopes against the *C. difficile* toxins, often by limiting the toxin domain repertoire, e.g., employing the CROPs domains to target potential receptor binding sites (8, 10). This might not be enough, however, as it has been shown that toxins with truncated or deleted CROP domains can still intoxicate the cells (34–36). The fact that, at least for TcdB, most of the identified receptor sites lie outside of the CROPs has led to more expansive screening of other toxin domains, as well as the full toxins, as vaccine antigens. The epitope distribution and the pattern of neutralization mechanisms described here reflect and strengthen the observations from the recent study on the broad panel of anti-toxin nanobodies (37), which used full-length toxins for immunization of alpacas.

Here, we show that functional, neutralizing antibodies can be elicited in mice by the full-length toxins, even with low levels of mutation or chemical modification of TcdB. The high affinities for the toxins exhibited by several of the murine mAbs, paired with their ability to neutralize TcdA and TcdB in cell culture and protect against toxin-mediated depolarization, suggest their potential to be effective *in vivo*, warranting further investigation in animal models of CDI. Future work will help determine if such potently neutralizing mAbs, as described herein, could be capable of preventing or lessening symptoms in the context of CDI, reducing the severity of disease.

## MATERIALS AND METHODS

### Cloning, expression, and purification of toxins, toxoids, and monoclonal antibodies (Pfizer)

Cloning, expression, and purification of genetically and chemically modified *C. difficile* toxoids A and B haves been previously described (15, 38). Wild-type TcdA and B genes were derived from the synthetic toxoid A and toxoid B genes (Blue Heron Biosciences), through reversion of the toxoid point mutations and the restriction sites used for sub-cloning. A *C. difficile* strain (VPI 11186) lacking endogenous toxin genes with a non-functional sporulation pathway was used as an expression host for the recombinant TcdA or TcdB gene.

#### Wild-type TcdA purification

Cell pellets of *C. difficile* expressing TcdA were homogenized and passed through a microfluidizer to lyse. After centrifugation, the soluble fraction of the cell lysate containing wild-type TcdA was applied onto a Reactive Red 120-Agarose (Sigma, catalog # R6143) dye affinity chromatography column equilibrated with 20 mM Bis-Tris, pH 6.5. Wild-type TcdA was eluted with a 0%–100% linear gradient of 20 mM Bis-Tris, pH 6.5, 3 M NaCl over 2.5 column volumes. Fractions were pooled and 300 mM sodium citrate added, and then, the sample was loadeding onto a Toyopearl Phenyl-650M hydrophobic column (Tosoh, catalog # 14783). The bound material was eluted step-wise with decreasing ionic strength buffer, and TcdA containing fractions were dialyzed against 20 mM MES, pH 6.5, 100 mM NaCl, 5 mM sodium phosphate. The dialyzed protein was loaded onto a Macro-Prep Ceramic Hydroxyapatite column (Type I, 40 µM, Bio-Rad Laboratories, catalog # 157-0041) equilibrated in 20 mM MES, pH 6.5, 100 mM NaCl, 5 mM sodium phosphate. The material was eluted using a 0%–100% linear gradient of this buffer with 400 mM sodium phosphate. TcdA containing fractions were pooled and extensively dialyzed against 20 mM Bis-Tris, pH 6.5, 150 mM NaCl, as described above. Protein concentration was estimated using UV absorbance, and the purity was assessed by SDS-PAGE.

#### Wild-type TcdB purification

Cell pellets were processed as described for TcdA. The soluble fraction of the cell lysate containing wild-type TcdB was applied onto a Toyopearl GigaCap Q-650M anion-exchange column (Tosoh, catalog #21855) equilibrated with 20 mM Bis-Tris, pH 6.5, 10 mM NaCl. The bound material was eluted using increasing gradients of this buffer with 700 mM NaCl. TcdB-containing fractions were pooled and loaded without any additional buffer exchange onto a Toyopearl Phenyl-650M hydrophobic column (Tosoh, catalog 14783). Bound material was eluted using with gradients of decreasing ionic strength, and TcdB-containing fractions were pooled, concentrated, and extensively dialyzed, as described above, against 20 mM. Bis-Tris pH 6.5, 150 mM NaCl. Protein concentration was estimated using UV absorbance, and purity was assessed by SDS-PAGE.

#### Hybridoma-based monoclonal antibody generation

The monoclonal antibodies (mAbs) used for part of the experiments described in this paper had been generated with the traditional hybridoma technology, developed by Cesar Milstein and George Koehler (39). Balb/C and Swiss Webster mice (female, 6–8 weeks old, Charles River, Kingston, NY or Harlan Laboratories, Haslett, MI) were immunized via the subcutaneous route on weeks 0 and 4 with 2 µg of TcdA (ListBio, CA, USA) or toxoid B (38), respectively, followed by an intraperitoneal boost (IP) at week 8. The antibody- producing B-cells in the splenocytes were fused with the mouse myeloma cell line PX63Ag8.653 (CRL-1580, ATCC, VA) using polyethylene glycol (PEG). The hybridomas were grown under hypoxanthine-aminopterin-thymidine (HAT) selection, subjected to limited dilution cloning to reach monoclonality followed by cryopreservation of the selected clones.

#### Monoclonal antibody production and purification

Selected mAbs for this study were also subjected to ascites fluid production, conducted by Covance under IACUC agreed protocols. For these productions, male severe combined immunodeficiency (SCID) mice (Taconic, NY) were used to minimize the mouse IgG background. IgG concentration was determined either by ELISA or by BioLayer Interferometry (BLI) using Fv biosensors (Fortebio, Molecular Devices, CA). Antibodies in the ascites fluid were purified either by affinity chromatography (protein G; mCDIFA-230-2, mCDIFA-248-25, mCDIFB-6-30, mCDIFB-56-15) or in a two-step process combining ammonium sulfate precipitation and ion-exchange chromatography (DEAE column; mCDIFA-60-22, mCDIFA-184-9, mCDIFA-205-7, mCDIFB-8-26). All antibody solutions were subjected to dialysis into final formulation buffer PBS, pH 7.2 either with or without 50% glycerol as preservative.

### Toxin neutralizing activity measurement

The ability of mAbs to neutralize the cytotoxicity of *C. difficile* toxins TcdA or TcdB was measured by toxin neutralization assay (TNA). African green monkey kidney Vero cells (epithelial cells, CCL-81, ATCC) were cultivated under standard conditions. For assays, cells were seeded into 96-well tissue culture plates at a density of 5 × 10^4^ cells per well and incubated overnight in a 37°C humidified incubator at 5% CO_2_. On the following day, appropriate serial dilutions of the mAbs were mixed with a fixed amount (4 × IC_50_) of wild-type toxins A or B. The toxins were purified from a *C. difficile* strain (ATCC BAA-1382) in which the toxin sequences were derived from strain 630 (VR-DRR-10452). After incubation at 37°C for 90 min, the mAb/toxin mixture was added to the Vero cell monolayer and incubated for 72 hrs in a humidified, 37°C/5% CO_2_ incubator. CellTiter Glo substrate (Promega, Madison, WI) was then added to wells to measure the adenosine triphosphate (ATP) levels present in the cells to indicate cell viability. CellTiter Glo activity was measured as Relative Luminescence Units (RLU) in a SpectraMax Luminescence plate reader (Molecular Device). The measured ATP levels and inferred cell viability are directly proportional to the level of neutralization, and thus, the amount of neutralizing antibody present in the sample. As controls in each assay plate, wells with toxin alone and no antibody (Toxin Only) and wells with media alone (Cell Only) were incorporated to establish the minimal and maximal cell viability respectively. Readings for experimental and Toxin Only wells were normalized to the Cell Only control to assess relative viability. Analysis was performed in GraphPad Prism by least squares fit of the *log(agonist) vs response—variable slope (four parameters*) model, and results were expressed as the EC_50_, which is defined as the concentration of antibody that increases viability to 50% between baseline and maximum protection. For mAb mCDIFA-248-25, the EC_50_ value was extrapolated from the available points since full titration could not be observed.

### Isothermal titration calorimetry

#### ITC Sample preparation

Both wild-type toxins (purified as described in the current manuscript) and corresponding toxoids (15) were used in the experiments in order to determine if genetic modifications and EDC treatment had affected epitope integrity. All proteins and antibodies were either extensively dialyzed or buffer exchanged into 1× PBS and centrifuged to remove any insoluble particles or aggregates. Concentrations were determined spectrophotometrically using extinction coefficients of 1.4 (mg/mL)^−1^ cm^−1^ for the antibodies, 1.3 (mg/mL)^−1^ cm^−1^ for TcdA, and 1.067 (mg/mL)^−1^ cm^−1^ for TcdB. Additionally, mCDIFA-60-22 required processing into Fab fragments using Pierce’s mouse IgG1 Fab and Fab_2_ Preparation kit (Pierce 44980).

#### ITC experimental setup, data analysis, and visualization

All binding studies reported here were performed on the VP-ITC or PEAQ-Micro-ITC (Malvern Panalytical). For the titrations, toxin concentrations were 5 µM, and the antibodies were typically 25 µM (equivalent of 50 µM of Fab fragments). To evaluate the binding of mCDIFA-60-22, the concentration of the toxin was adjusted to 2 µM and the concentration of the mAb was adjusted to 120 µM to account for multiple binding sites. In cases where the toxin could not be saturated, even under described conditions, a reverse titration was employed with 10 µM of the Fab fragments in the sample cell titrated with 20–40 µM of TcdA. Analysis was performed using Origin software (Malvern). The fitting was performed using the “single class of binding sites” model to obtain the stoichiometry of binding (N), dissociation equilibrium binding constant (*K*_d_ (M^−1^)), enthalpy change upon binding (Δ*H* (kcal mol^−1^)), and entropic contribution to the free energy change upon binding (-TΔS, where Δ*S* is the entropy upon binding (kcal mol^−1^ K^−1^) and *T* is experimental temperature in degrees Kelvin [°K]).

### Mapping of the antibody-binding epitopes using HDX-MS

#### Sample preparation

Stock solutions of the antigens (wild-type toxin A for TcdA-specific antibody mapping and EDC-treated genetic toxoid B for TcdB-specific antibody mapping) and antigen-antibody complexes were prepared at 8–9 µM antigen concentration in 1× PBS. The majority of the antibodies were combined with the corresponding antigens at 10% molar excess of the Fab fragment concentrations over the antigen concentration. mCDIFA-60-22 Fab fragments were combined with TcdA at 4:1 molar ratio of the Fab fragments to the antigen.

Hydrogen-deuterium exchange experiments were set up automatically using the Leap PAL HDX robot. Sample aliquots (5 µL) were combined with 20 µL of 1× PBS in 90% D_2_O to initiate the exchange (20 µL of H_2_O-based 1× PBS during initial proteolytic digest mapping). After 2, 6, 20, 60, and 180 min of exchange (10 hrs and 30 hrs for mCDIFA-60-22 which recognizes epitopes in the structurally stable CROP domain, where very little exchange is taking place), the samples were transferred into 75 µL of a quenching buffer. Two sets of quenching conditions (producing slightly different proteolytic digest maps) were employed: 3.2 M guanidine-HCl, 0.67% formic acid, 0.5 mg/mL pepsin, at 2°C and 8.0 M guanidine—HCl, 0.67% formic acid at 15°C. Samples quenched at the higher guanidine-HCl concentration were additionally diluted 1:1 with 1.0 mg/mL pepsin in 0.5% formic acid. The samples were then injected onto the HDX system with a pepsin column (Waters Enzymate, catalog number 186007233) included in-line to help with digestion.

Proteolytic peptides were separated on a 50 × 1 mm Hypersil Gold C18 column using a linear gradient of either 12%–40% or 15%–35% acetonitrile, 0.1% formic acid and analyzed on OrbiTrap Fusion Lumos or OrbiTrap QExactive Plus mass spectrometers (Thermo Scientific) operated in the positive mode. Proteolytic peptides were identified through a search of MS/MS data obtained for the non-deuterated samples using Sequest HT or BioPharmaFinder 3.1 (Thermo Scientific). Deuterium accumulation at individual time points was analyzed using HDExaminer 3.2 (Sierra Analytics). Antibody-binding epitopes were identified from the reduced deuterium accumulation on proteolytic peptides derived from the antibody-bound toxins, as compared to the same peptides derived from the control experiment with unbound toxins.

### Cloning, expression, and purification of recombinant toxins and antibody fragments (Vanderbilt)

Recombinant toxins were expressed and purified from plasmids in a *Bacillus megaterium* system as previously described: TcdA_1–2710_ (pBL282), TcdA_1–1832_ (pBL515), TcdB_1–2366_ (pBL377), TcdB_1–1810_ (pBL832) (13, 21, 40). These preparations were used in negative stain electron microscopy experiments, cell binding assays, and cell depolarization assays.

Purified monoclonal antibodies (1 mg total) were digested with a 1:100 mass ratio of papain:mAb. Reactions were incubated for 1 h in a 37°C water bath, quenched with 20 mM of freshly made iodoacetamide, and dialyzed against 20 mM Tris pH 8.0. Dialyzed samples were applied to a 1 mL HiTrap Q HP column (Cytiva) equilibrated in 20 mM Tris pH 8.0, and the Fabs were separated from Fc through increasing step-elutions of NaCl (50/100/200 mM). SDS-PAGE was performed to confirm Fab-containing fractions, and final concentrations were determined by *A*_280_ (*E*_0.1%_ 1.4, MW 50,000 Da).

### Negative-stain electron microscopy of toxin-antibody complexes

Reactions containing 100 nM of purified toxins TcdA_1–1832_, TcdA_1–2710_, or TcdB_1–1810_ were set up with 2x molar excess of antibody Fab fragment in 20 nM Tris pH 8.0, 100 mM NaCl, incubated for 30 min at room temperature, and then diluted 5x in the same buffer immediately before grid preparation. Diluted samples (3 µL) were applied to glow-discharged, carbon-coated copper grids (Electron Microscopy Sciences, Cu-400) for negative staining with 0.75% uranyl formate (41). Micrographs of the toxin-Fab complexes were collected at 62,000x magnification (1.75 Å/pixel) on a Tecnai TF20 (200 keV; ThermoFisher) transmission electron microscope equipped with a Gatan 4Kx4K CCD camera, using the Serial EM software suite (42). Micrographs were processed through automatic particle picking (reference-based; box size 250 × 250 pixels), and two-dimensional alignment and class averaging was performed with either XMIPP3 in the Scipion software suite (43, 44) or RELION (45). For the mCDIF56-15 Fab, samples were diluted in SEC buffer, applied to glow-discharged (Pelco EasiGlow, 0.19 mBar, −15 mA, 20 s) copper grids with a thin carbon film (CF300-Cu-UL, EMS), washed with water, and stained with 2% uranyl acetate. Grids were imaged at 50,000× magnification (2.087 Å/pix) using a Tecnai TF20 transmission electron microscope (200 keV; ThermoFisher) equipped with a OneView camera (Gatan). Data were collected using Serial EM and processed with RELION (45). In part, structural biology software used in this project was compiled and configured by SBGrid (46).

### Mammalian cell culture

A549 (ATCC CCL-185) and Vero (ATCC CCL-81) cells were maintained in Dulbecco’s modified Eagle’s medium (DMEM, Corning) supplemented with 10% fetal bovine serum (FBS, Atlanta Biologicals). Caco-2 cells (ATCC HTB-37) were maintained in Eagle’s minimum essential medium (EMEM, Corning) supplemented with 10% FBS. HT29 cells (ATCC HTB-38) were cultured in McCoy’s 5 a Medium modified with L-glutamine (Corning) and 10% FBS.

### Toxin cell-binding assays

A549, Caco-2, or Vero cells were seeded at a density of 250,000 cells/mL in 6-well plates (Caco-2) or 10 cm dishes (A549 and Vero) and incubated at 37°C, 5% CO_2_ for 48 hrs. Purified toxin TcdA or TcdB (10 nM) was pre-incubated with a 10-fold excess of mAb or Fab for 20 min at room temperature. Toxin-antibody complexes were added to confluent cell monolayers that were prechilled at 4°C for 1 hr. Controls included cells that did not receive toxin and cells that received 10 nM toxin only. Toxins and toxin-antibody complexes were incubated at 4°C for 1 hr, followed by a brief incubation at 37°C for 5–10 min. Media was removed from cells, and monolayers were washed two times with chilled PBS. Cells were dislodged in PBS with a cell scraper and pelleted at 1,000 × *g* for 5 min at 4°C. Cell pellets were suspended in sucrose lysis buffer (10 mM Tris pH 7.4, 250 mM sucrose, 3 mM imidazole) with 1:100 vol protease inhibitor cocktail (P8340, Sigma) and then lysed by sonication. Cell debris was removed by centrifugation at 1,500 × *g* for 15 min at 4°C. Twenty-five nanograms total protein of each sample lysate was diluted with Laemmli buffer, heated at 95°C for 5 min, and loaded onto 4%–20% gradient Mini-PROTEAN TGX gels (Bio-Rad). Proteins were transferred in Tris-glycine-methanol buffer to PVDF membranes at 100 V for 1 hr and blocked with 5% milk in Tris-buffered saline with 0.1% Tween 20 (TBS-T) for 1 h at room temperature. Primary antibodies against TcdA (1:1,000, NB600-1066, Novus Biologicals), TcdB (20B3; 43), and GAPDH (1:5,000, 14C10, Cell Signaling) were diluted in 5% milk-TBS-T and incubated with the membranes overnight at 4°C. Membranes were washed three times with TBS-T and then incubated with secondary antibodies anti-mouse-HRP (1:5,000, 7076S, Cell Signaling) or anti-rabbit-HRP (1:5,000, 7074S, Cell Signaling) for 1 hr at room temperature. Membranes were washed three times with TBS-T, and HRP was detected using ECL Western blotting substrate (32106, Pierce) or Immobilon Western chemiluminescent HRP substrate (WBKLS0500, Millipore). Each binding experiment was repeated three times. Densitometry quantification was performed in Fiji.

### Cellular depolarization assays

#### TcdA at acidic pH

HT29 cells were plated in black, clear-bottom, tissue-culture treated, 96-well plates (Corning) at a density of 15,000 cells per well and incubated at 37°C, 5% CO_2_ for 48 hrs. Medium was aspirated from the wells, and the cells were washed twice with 100 µL of Hanks’ Balanced Salt Solution (HBSS, with calcium and magnesium, without phenol red, pH adjusted to 5.0 with HCl; Corning). 100 µL of a freshly made solution of 5 µM DiBAC_4_(3) (Bis-(1,3-dibutylbarbituric acid) trimethine oxonol; Thermo B438) in HBSS was added to each well and incubated at 37°C for 30 min. At the same time, toxin TcdA was pre-incubated with a 10-fold excess of mAb in HBSS pH 5.0 for 30 min at room temperature. After incubation with the dye, the cells were washed twice with HBSS pH 5.0, then 90 µL of HBSS was added to each well. 10 µL of each toxin-antibody mixture was added to the cells, and the plate was read immediately in a Cytation plate reader (Biotek), 490/516 em/ex (10 nm slits), every 5 min for 2 hr. Three experimental replicates were performed for each condition, with technical replicates averaged and background corrected by subtraction of a no-toxin control.

#### TcdB at neutral pH

A549 cells were plated in black, clear-bottom, tissue-culture treat, 96-well plates (Corning) at a density of 15,000 cells per well and incubated at 37°C, 5% CO_2_ for 26 hr. The protocol followed the same as for TcdA at low pH, but with HBSS pH 7.2 used for the toxin-antibody dilutions and the final HBSS volume added to cells before intoxication.

### Cryo-electron microscopy of toxoid A-Fab mCDIFA-248-25 complex

#### Preparation of toxoid A- Fab mCDIFA-248-25 complex

Inactivated cross-linked *C. difficile* toxoid A (TxdA) was expressed and purified as previously described (15). mCDIFA-248-25 Fab was prepared as previously described in Materials and Methods. Fab mCDIFA-248-25 was incubated with toxoid A at a 2:1 molar excess for 30 min on ice, concentrated, and loaded onto a Superose 6 Increase 10/300 Gl column equilibrated in 25 mM Tris, pH 7.5, 150 mM NaCl. Fractions were analyzed by SDS-PAGE and pooled based on co-elution of toxoid A with Fab mCDIFA-248-25.

#### Grid preparation and data acquisition

The purified toxoid A-Fab mCDIFA-248-25 complex was diluted to 0.125 mg/mL, applied to Quantifoil R1.2/1.3 200 mesh Au grids, and vitrified using a Vitrobot Mark IV (ThermoFisher Scientific). Dose fractionated movies were acquired using SerialEM (42) from a Titan Krios G2 transmission electron microscope (ThermoFisher Scientific) operating at 300 keV equipped with a Gatan K2 Summit direct electron detector, in super-resolution mode with a pixel size of 0.543 Å/pixel over a defocus range of −1.5 to −2.5 µm at a dose rate of 4.35 e-/Å^2^/s, with 12 sec exposures for a total of 48 frames.

#### CryoEM data processing

Individual frames from each of the 2,511 raw movies were aligned using MotionCor2 v1.0.5 (47) and CTF estimated using CTFFIND4 (48). The initial template-free particle picking was performed using WARP 1.0.9 (49) and provided 180,313 picked particles. The coordinates for these particles were integrated into RELION-3.1.0 (50) to generate initial 2D class average templates for RELION reference-based auto-picking, which picked 527,742 particles. The rest of the cryoEM data processing was done using RELION-3.1.0. The particles picked by RELION were extracted, binned by 2, and classified into one hundred100 2D class averages after 50 iterations. Particles from 2D class averages with clear secondary structure features were selected and unbinned for a second round of 2D classification. From the second round of 2D classification, 475,506 particles from 7 good classes were selected for *ab initio* 3D reconstruction and 3D classification. The *ab initio* 3D reconstruction was performed using stochastic gradient decent (SGD) procedure (51) with the CTF-modulation options disabled and the resulting 3D structure was used as the initial model for 3D classification into 8 classes with spherical mask diameter of 400 Å and without applied- symmetry (C1). Particles from two class averages with clear α-helical and β-strand features containing a total of 212,265 particles were selected for 3D auto-refinement.

The first round of 3D auto-refinement using the best 3D class average low-pass filtered to 60 Å as the initial model, a mask generated using the best 3D class, particles from the selected 2D classes and without symmetry resolved a 3.9 Å resolution structure after post-processing. Two iterations of CTF refinement and particle polishing followed by 3D auto-refinement and postprocessing generated a final cryoEM map of 3.2 Å resolution based on the 0.143 criteria from the “gold-standard” FSC curve. The final cryoEM map was flipped since the direction of the α-helices turned in the opposite direction. Local resolution analysis was calculated using ResMap (52). The cryoEM map was also sharpened using the *phenix.auto_sharpen* command in the Phenix software package version 3594 (53).

#### Atomic modeling and model refinement

The structure of TcdA (21) (PDB: 4R04) containing the glucosyltransferase domain (GTD), autoprocessing domain (APD), and the delivery domain was docked into the cryoEM structure. The atomic model was built with minor refinements in COOT (54) guided by the docked structure and EM densities for bulky side chains. Since the C-terminal combined repetitive oligopeptides (CROPS) wereas modeled *de novo* with the help of a homology model, the sharpened map was used to assist in the modeling of this region as the EM densities for the CROPS isare slightly worse compared to the rest of the structure.

## Data Availability

The final cryoEM density map and model of TcdA in complex with mCDIFA-248-25 Fab are deposited in the Electron Microscopy Data Bank (EMDB) under accession code EMD-48707 and Protein Data Bank (PDB) under accession code 9MX1, respectively. Additionally, data from this finding are available from the corresponding author upon request.

## SUPPLEMENTAL MATERIAL

Supplemental material iai.00139-25-s0001.docx Fig. S1 to S9; Tables S1 and S2.

ASM does not own the copyrights to Supplemental Material that may be linked to, or accessed through, an article. The authors have granted ASM a non-exclusive, world-wide license to publish the Supplemental Material files. Please contact the corresponding author directly for reuse.
