# Supplementary material for: Mouse monoclonal antibodies against Clostridioides difficile toxins TcdA and TcdB target diverse epitopes for neutralization
Source: Infect Immun. 2025 Aug 22;93(10):e00139-25. doi: 10.1128/iai.00139-25 (PMC12519798; doi:10.1128/iai.00139-25)
Supplement: Supplemental material — Fig. S1 to S9; Tables S1 and S2. [file iai.00139-25-s0001.docx]

**Supplemental Figures**

**Figure S1. Representative deuterium uptake plots used to map mCDIFA-60-22 epitopes.**

*Deuterium uptake plots show % deuteration as a function of incubation time in D_2_O. Each peptide is identified by its position within amino acid sequence of TxdA, with specific amino acid sequences shown in plot titles. Numbers in parenthesis indicate only respective peptide’s number in the HDExaminer project file used to analyze the data. Green symbols – high confidence results, yellow symbols – medium confidence results (as defined by HDExaminer). Lines are drawn solely to guide the eye.*


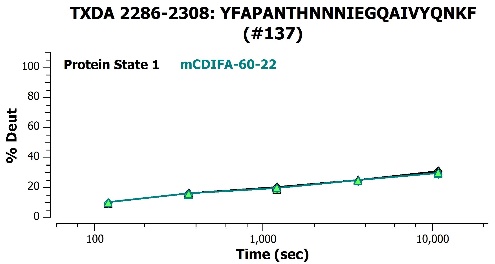

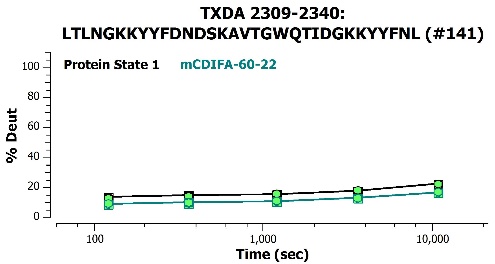

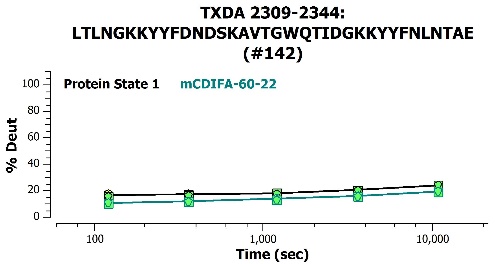

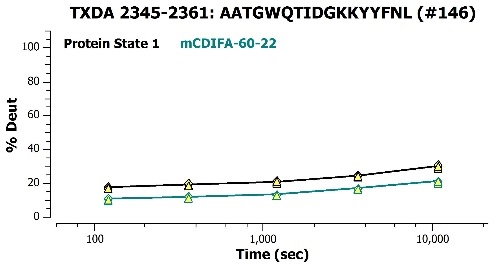

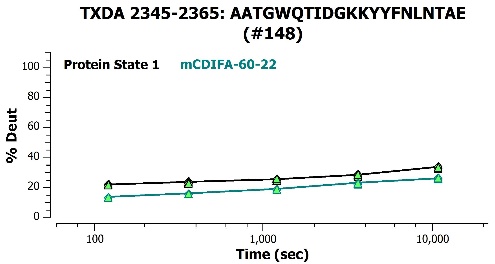

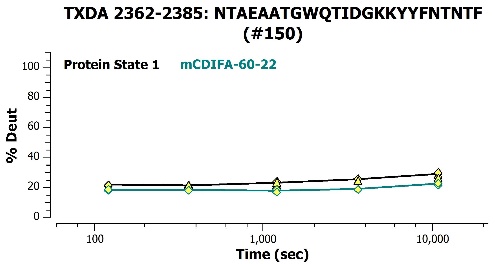

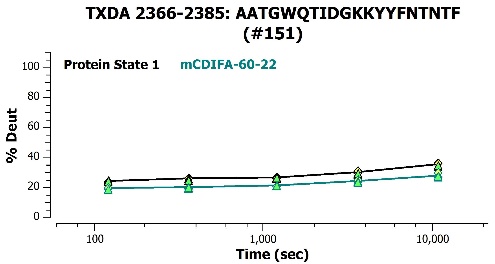

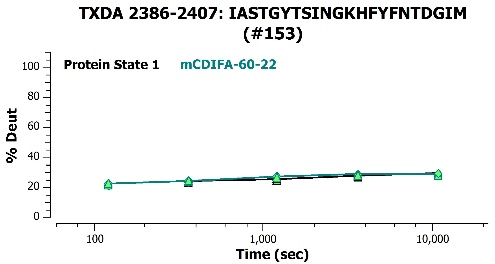

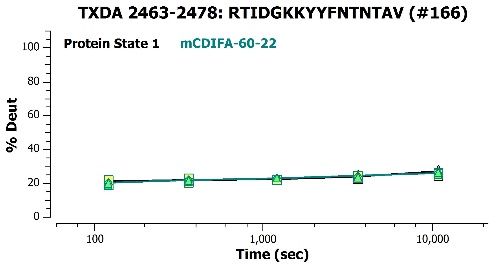

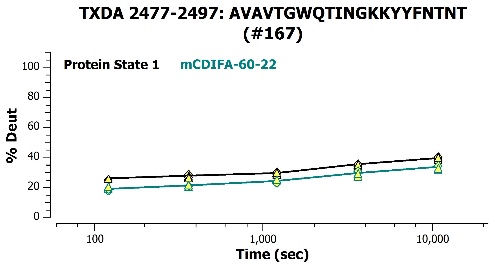

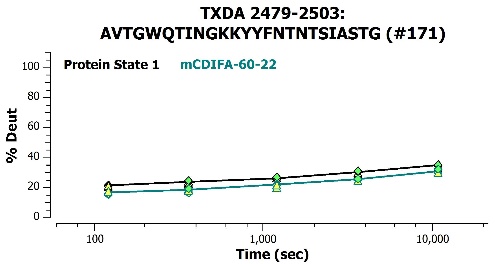

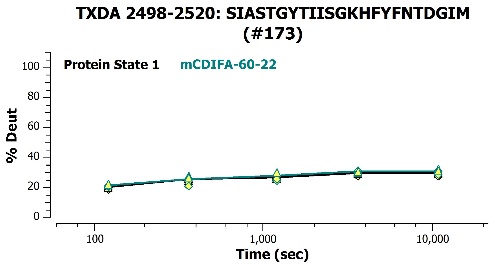

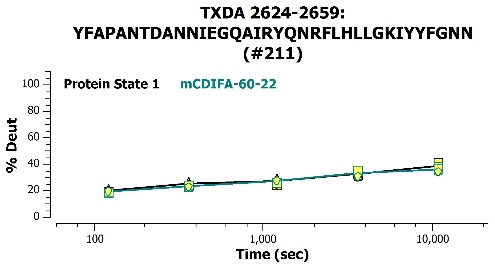
**
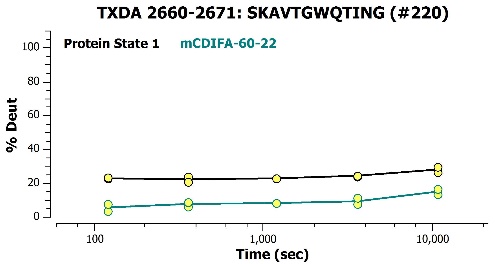

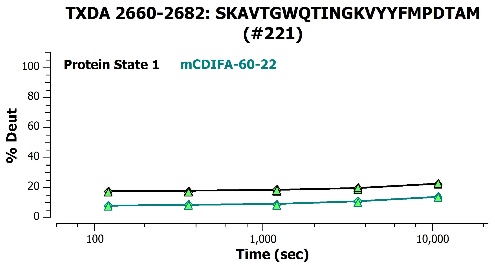

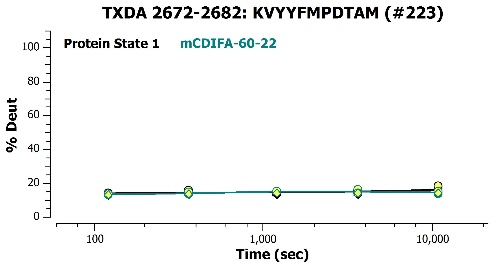
**

**Figure S2. Representative deuterium uptake plots used to map mCDIFA-184-9 epitope.**

Symbols and labeling conventions are the same, as Figure S2.

**
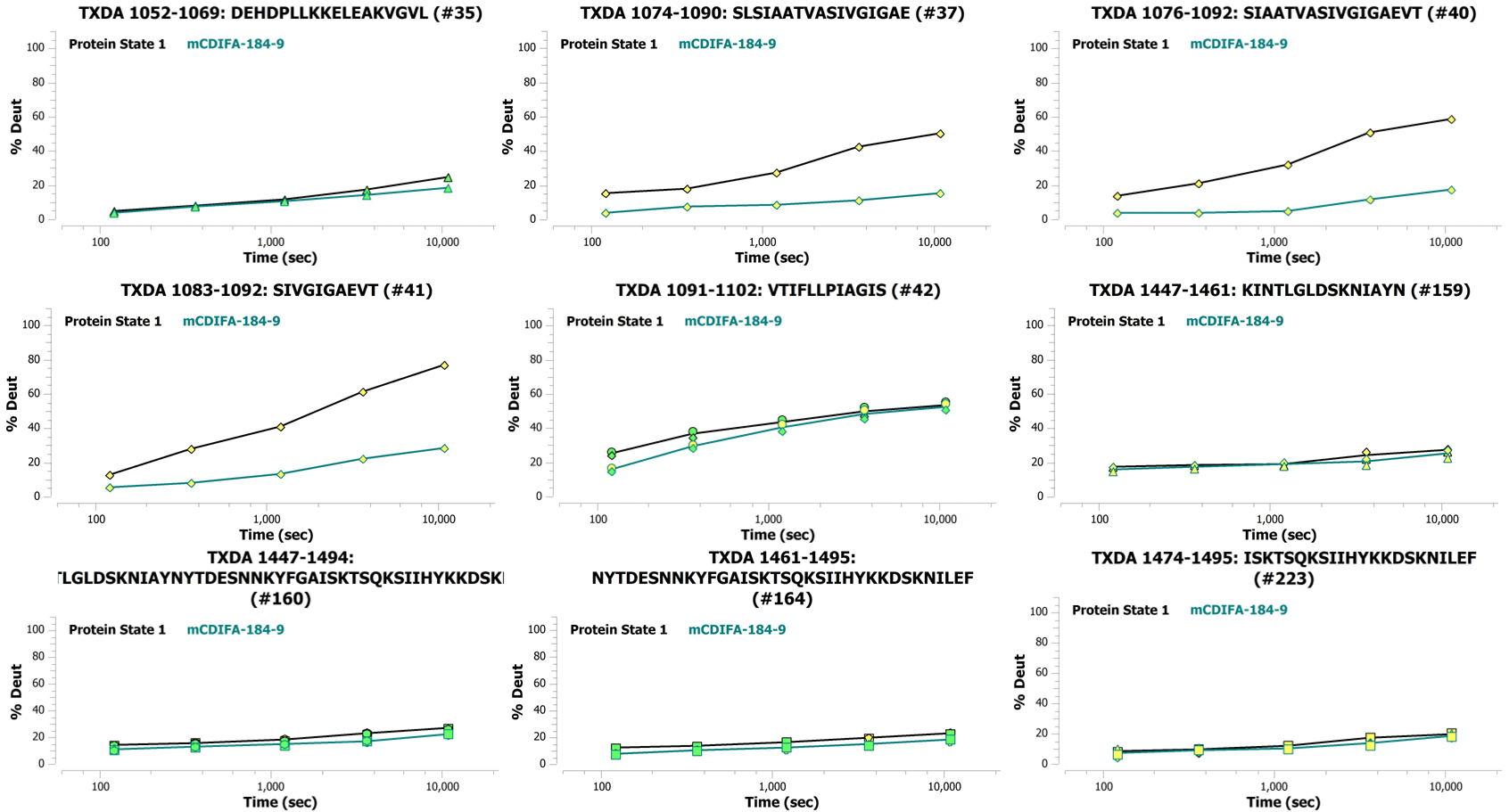
**

**Figure S3.** **Representative deuterium uptake plots used to map mCDIFA-205-7 epitope.**

Symbols and labeling conventions are the same, as Figure S2.

**
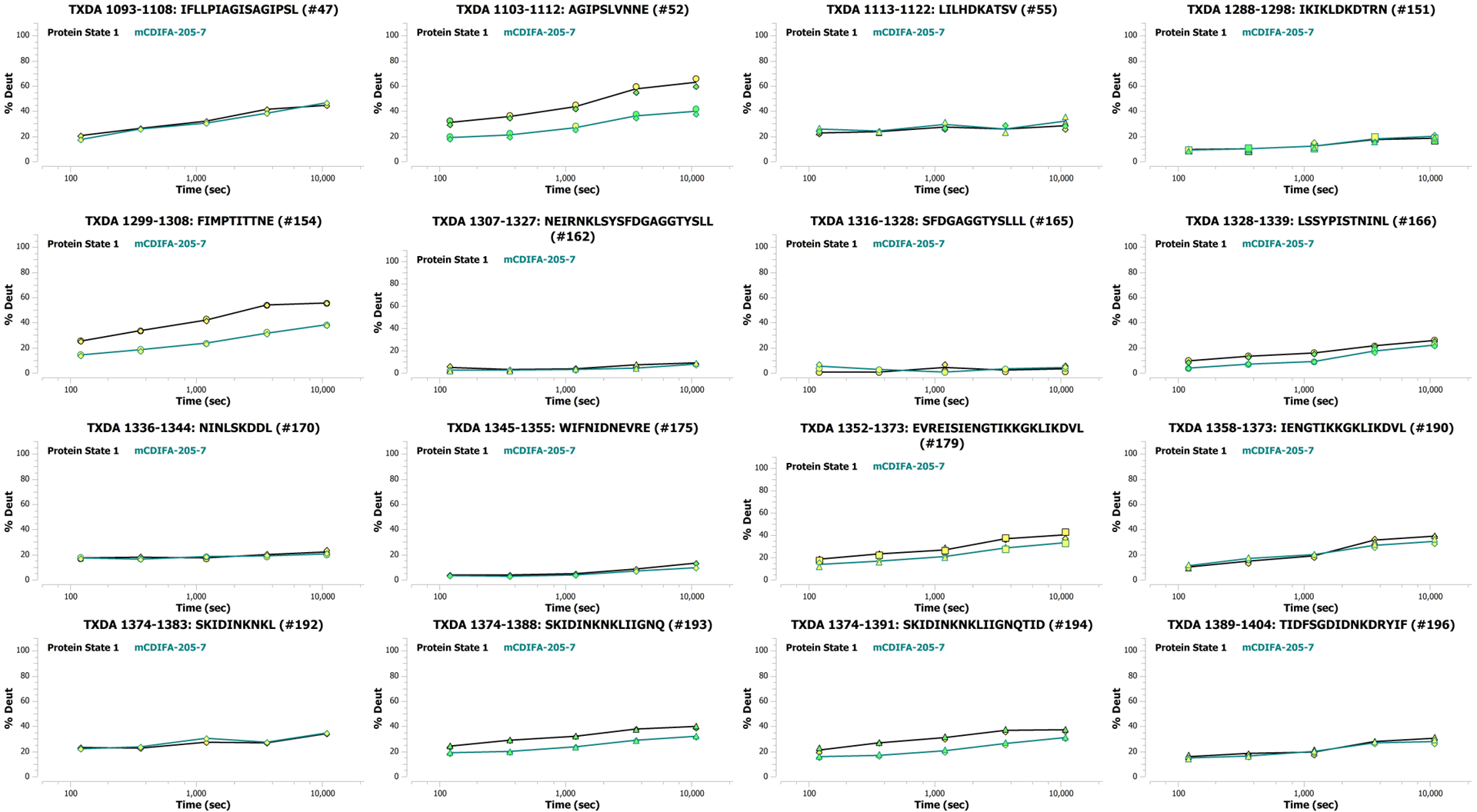
**

**Figure S4. Representative deuterium uptake plots used to map mCDIFA-230-2 epitope.**

Symbols and labeling conventions are the same, as Figure S2


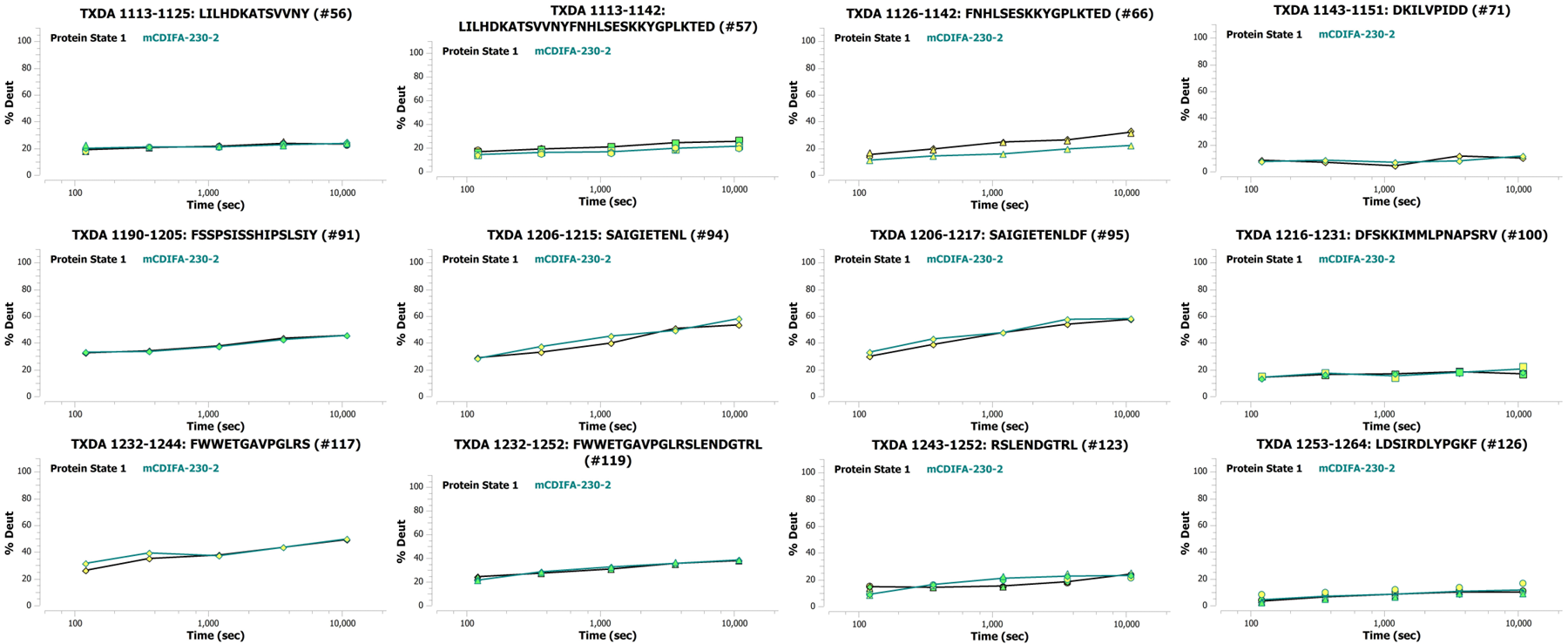


**Figure S5.** **Representative deuterium uptake plots used to map mCDIFA-248-25 epitope.**

Symbols and labeling conventions are the same, as Figure S2.

**
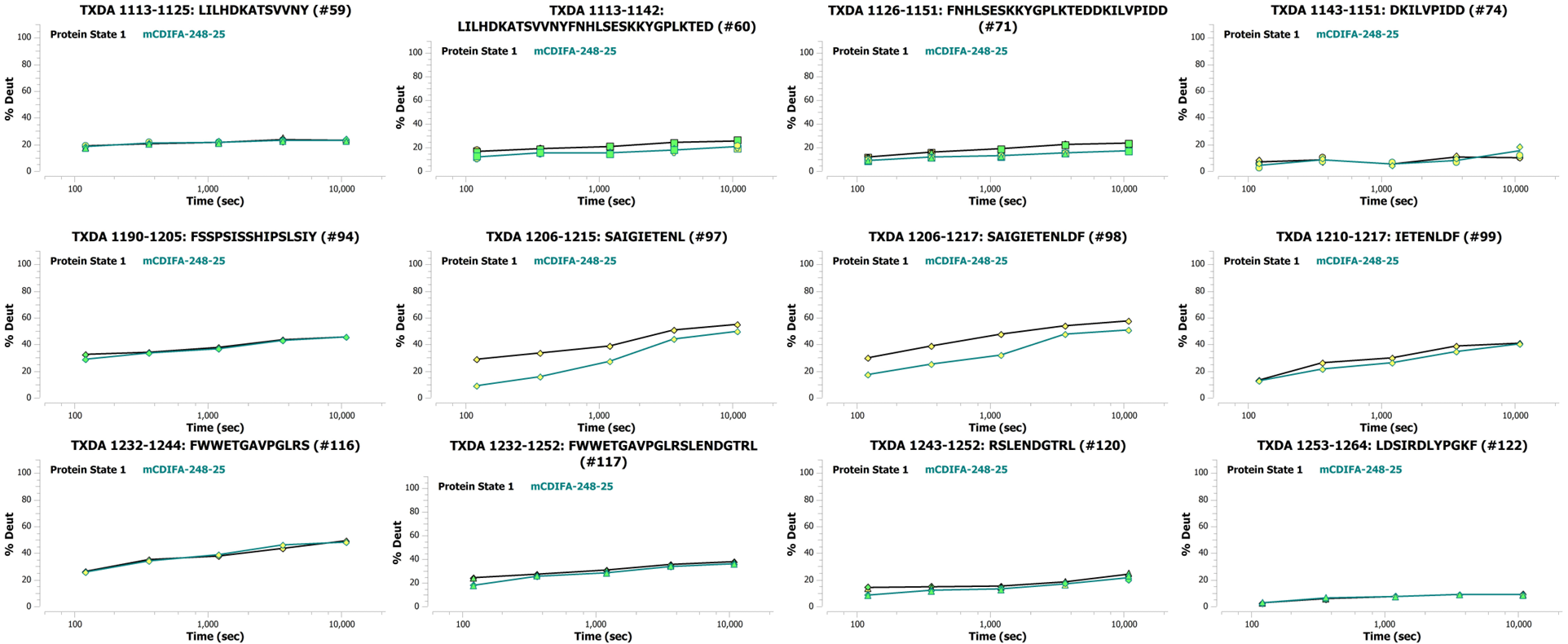
**

**Figure S6. Representative deuterium uptake plots used to map mCDIFB-8-26 epitope.**

Symbols and labeling conventions are the same, as Figure S2.

**
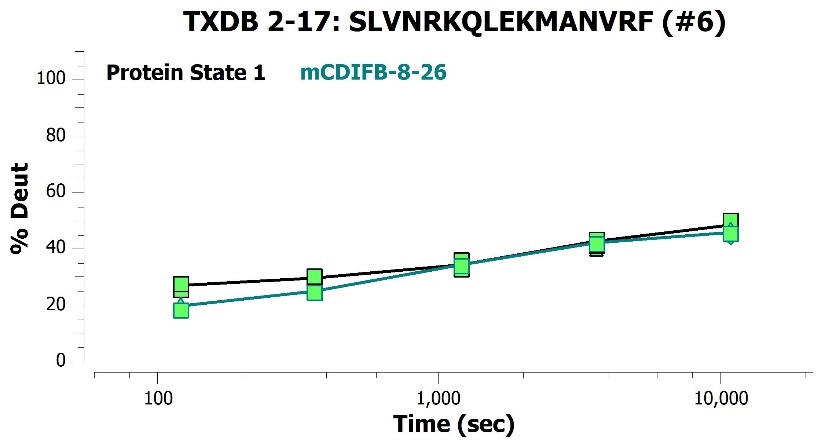

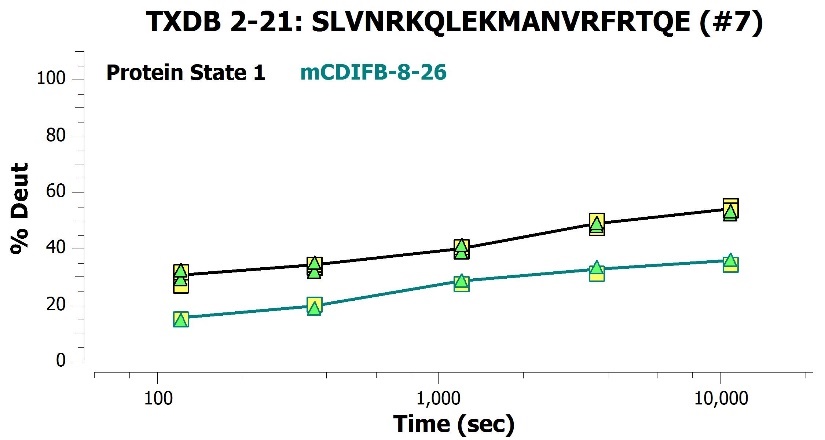

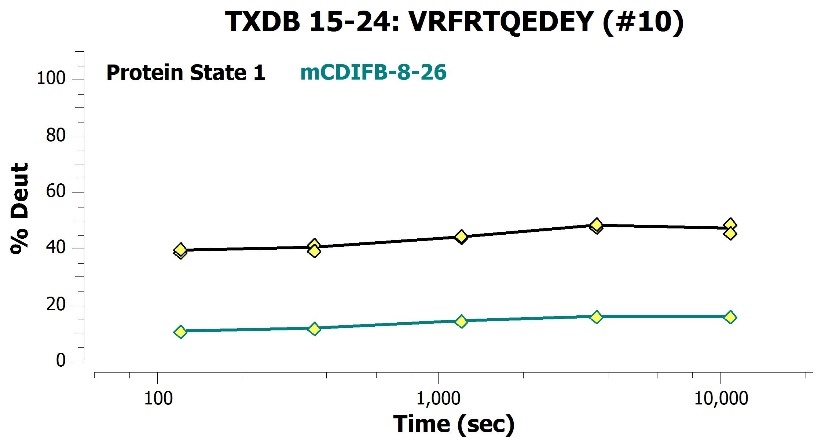

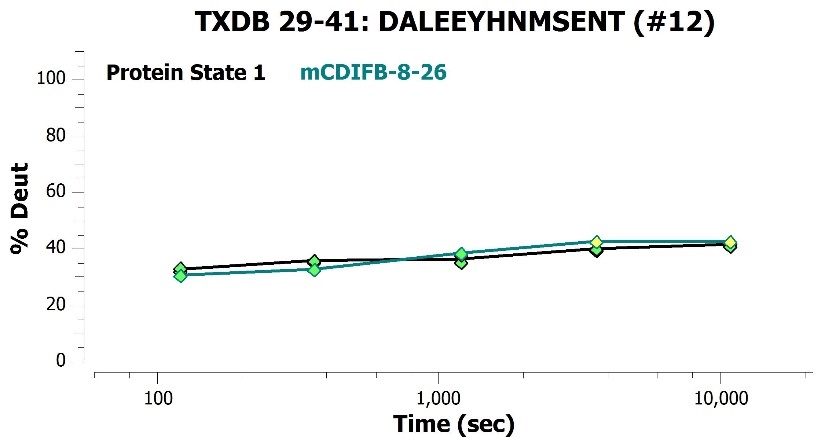
**

**Figure S7. Representative deuterium uptake plots used to map mCDIFB-6-30 epitope.**

Symbols and labeling conventions are the same, as Figure S2.

**
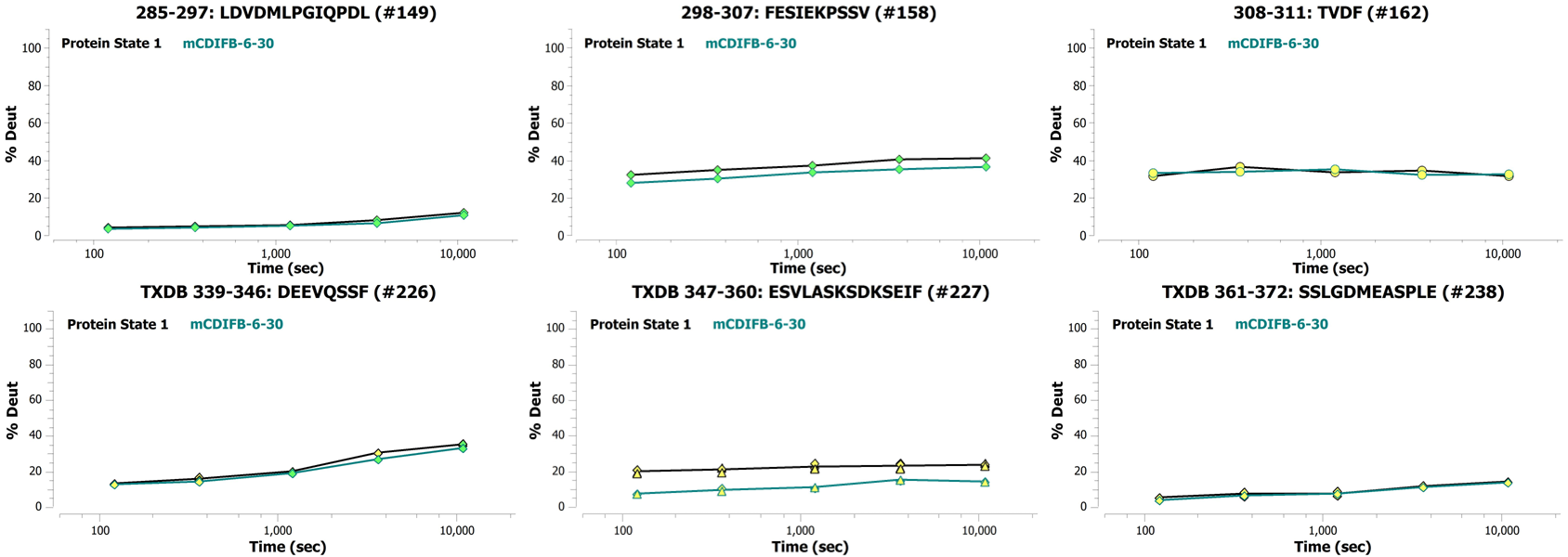
**

**Figure S8.** **Representative deuterium uptake plots used to map mCDIFB-56-15 epitopes.**

Symbols and labeling conventions are the same, as Figure S2.

**
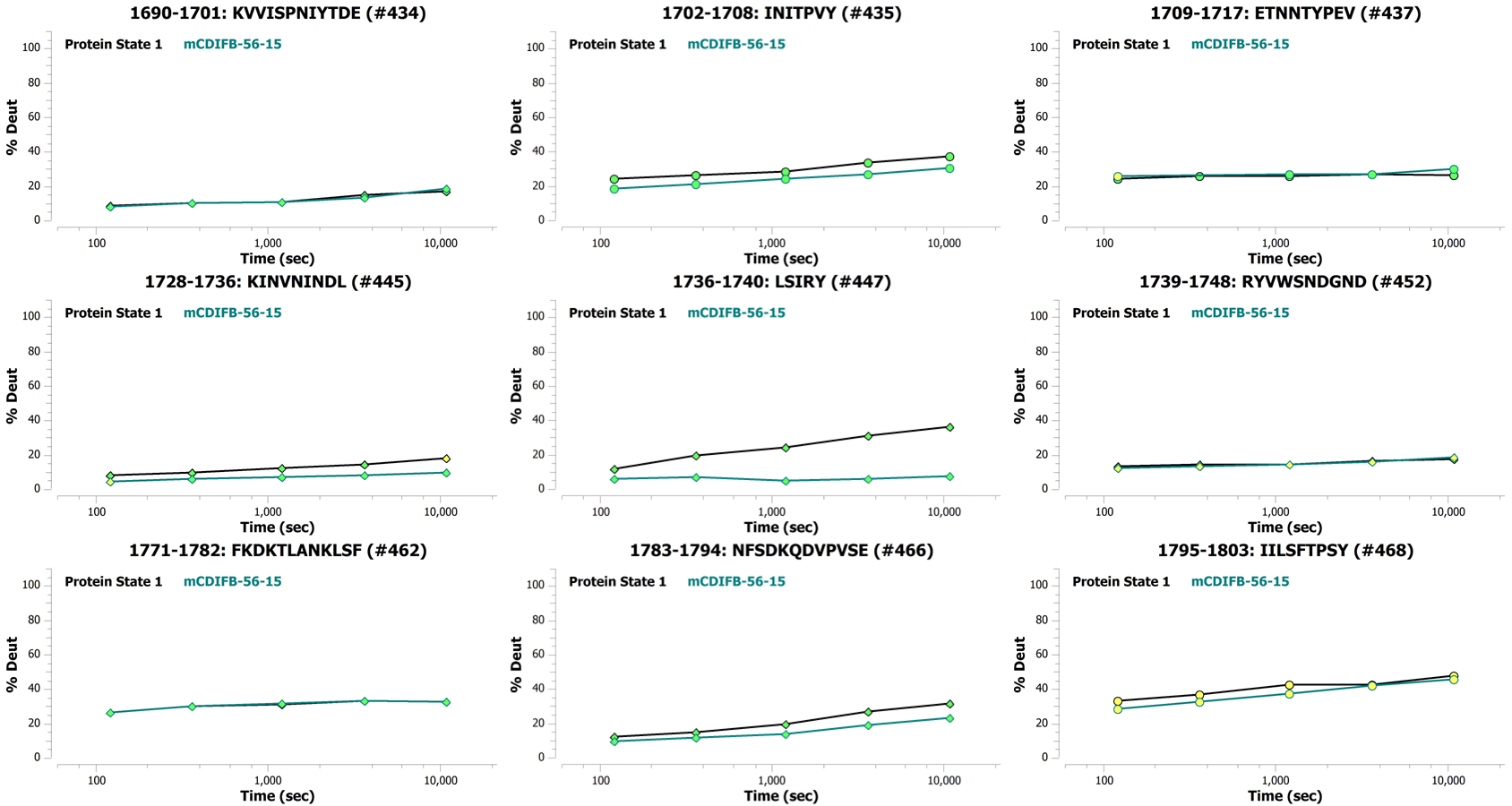
**

**Figure S9. Cryo-EM data processing workflow.** (A) Cryo-EM data processing workflow. (B) The FSC curve and (C) the model to map FSC curve showing the resolution of the structure based on the gold-standard FSC_0.143_ criterion.

**
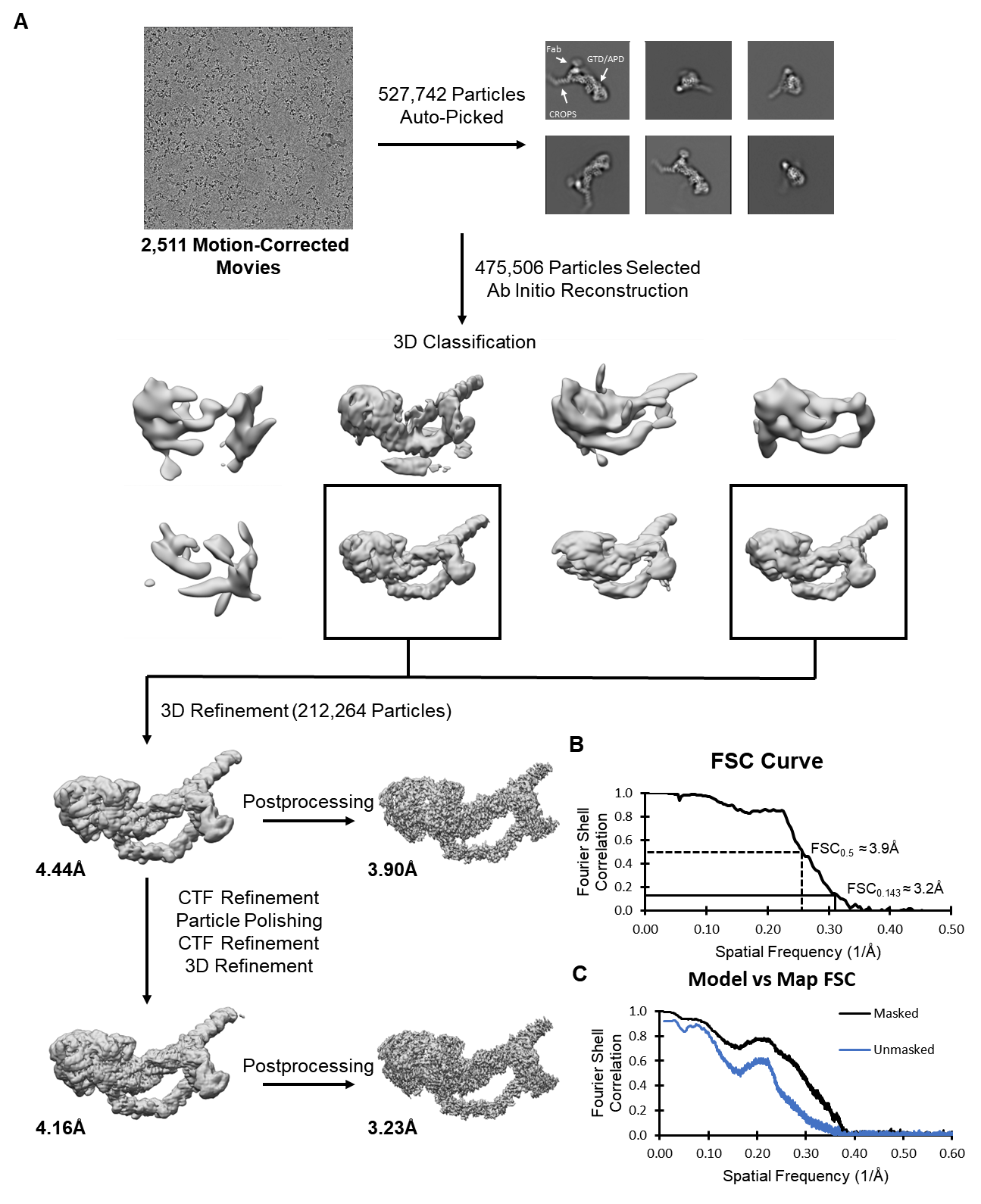
**

**Supplemental Table 1.**

**Data Collection, Processing and Refinement Statistics**

EMDB: #####

PDB: ####

**Data Collection and Processing**

Magnification 130,000x

Voltage (kV) 300

Total Electron Exposure (e^-^/Å^2^) 52.2

Defocus Range (μm) -1.5 to -2.5

Pixel Size (Å)

Super-Resolution Mode 0.543

Movies Recorded 2,511

Symmetry Imposed C1

Initial Particle Images (no.) 527,742

Final Particle Images (no.) 212,265

Map Resolution at FSC=0.143 (Å) 3.23

**Refinement**

Map sharpening *B* factor (Å^2^) -62.25

Model composition in the

asymmetric unit

Non-hydrogen atoms 20,832

Protein residues 2,594

B factors (Å^2^)

Protein 37.0

R.M.S. Deviations

Bond lengths (Å) 0.004

Bond angles (°) 0.761

Validation

MolProbity score 1.63

Clashscore 4.34

Poor rotamer (%) 0.04

Ramachandran Plot

Favored (%) 93.62

Allowed (%) 6.38

Outliers (%) 0.00

**Supplemental Table 2.**

**Hydrogen bonds within the TcdA-mCDIFA-248-25 epitope (ePISA analysis)**

| **TcdA** | **Heavy chain** |
| --- | --- |
| I1210 | N31 |
| S1130 | Y100 |
| S1255 | Y103 |
| A1207 | Y103 |
| K1134 | Y103 |
| K1134 | Y100 |
| K1133 | Y100 |
| K1133 | G101 |
| K1133 | F102 |
| R1251 | P107 |
| K1134 | Y109 |
| D1258 | K57 (*salt bridge*) |
|  |  |
| **TcdA** | **Light chain** |
| A2176 | R238 |
| N1247 | V249 |
| N1247 | G250 |
| R1251 | Y311 |
